# High-quality genome assembly of *Impatiens noli-tangere* reveals key insights into α-linolenic acid biosynthesis and metabolic volatiles

**DOI:** 10.1093/hr/uhaf216

**Published:** 2025-08-22

**Authors:** Ning Xu, Fan Kang, Yanan Deng, Bin Xia, Yujia Yang, Shengyan Chen, Shuo Sun, Yulu Zhao, Miao He, Huiyan Gu, Yunwei Zhou

**Affiliations:** School of Forestry, Northeast Forestry University, Harbin, Heilongjiang, China; College of Landscape Architecture, Northeast Forestry University, Harbin, Heilongjiang, China; School of Forestry, Northeast Forestry University, Harbin, Heilongjiang, China; College of Landscape Architecture, Northeast Forestry University, Harbin, Heilongjiang, China; College of Landscape Architecture, Northeast Forestry University, Harbin, Heilongjiang, China; College of Landscape Architecture, Northeast Forestry University, Harbin, Heilongjiang, China; School of Forestry, Northeast Forestry University, Harbin, Heilongjiang, China; School of Forestry, Northeast Forestry University, Harbin, Heilongjiang, China; College of Landscape Architecture, Northeast Forestry University, Harbin, Heilongjiang, China; School of Forestry, Northeast Forestry University, Harbin, Heilongjiang, China; College of Horticulture, Jilin Agricultural University, Changchun, Jilin, China

## Abstract

*Impatiens noli-tangere* accumulates abundant α-linolenic acid (ALA) and its metabolic volatiles, which hold significant potential for applications in healthcare and agriculture. However, the genetic basis underlying their biosynthesis has not been systematically investigated. Here, we present a high-quality genome assembly for *I. noli-tangere* (614.46 Mb). Despite a high repetitive sequence content (70.46%), it avoided excessive expansion due to the efficient elimination of long terminal repeat retrotransposons. Phylogenomic analyses revealed that *I. noli-tangere* experienced two whole-genome duplication (WGD) events, with WGD-derived genes predominating in oil biosynthesis. Notably, *IntFAD3*, a WGD-duplicated fatty acid desaturase, was identified as a key seed-specific gene for ALA biosynthesis. Its regulation by the transcription factor *IntbZIP38* was functionally validated through yeast one-hybrid, luciferase, β-glucuronidase, and transgenic functional assays. Furthermore, (E)-2-hexenal, the predominant ALA-derived volatile in leaves, exhibited potent antifungal activity against *Botrytis cinerea* (minimum inhibitory concentration: 0.188 ml/l), with its biosynthesis linked to *Int13-HPL*. These findings provide genomic and functional insights into ALA biosynthesis and metabolic volatiles in *I. noli-tangere*, supporting its potential in sustainable agriculture and bioactive compound development.

## Introduction

The genus *Impatiens* comprises over 1000 species distributed across tropical, subtropical, and temperate regions [1]. Renowned for their vibrant flowers, distinctive morphology, and adaptability, these species are widely cultivated as ornamental plants, contributing significantly to horticultural and landscaping industries [2]. In the United States alone, *Impatiens* species contribute over $100 million annually in the horticultural market [3]. Beyond their ornamental appeal, *Impatiens* species harbor substantial potential in fields, such as food chemistry, plant protection, and healthcare, with more than 300 bioactive compounds identified across the genus [4, 5]. However, despite this remarkable chemodiversity, the biological roles and regulatory mechanisms of these metabolites remain poorly understood due to limited genomic and functional resources in the genus.

Among the numerous bioactive compounds reported in *Impatiens*, α-linolenic acid (ALA) has attracted particular attention due to its essential role in human health [6]. As an essential fatty acid, ALA cannot be synthesized by humans but exhibits significant bioactivities, including reducing cardiovascular disease risk, modulating inflammation, and supporting neurological functions [7, 8]. *I. noli-tangere* stands out within the genus for its high ALA content in seeds and leaves [9]. Notably, its seed oil contains ~31.6% ALA, surpassing the levels found in conventional oil crops like sunflower, sesame, and soybean [10–12]. This exceptional ALA content positions *I. noli-tangere* as a promising source for dietary supplements with broad implications for food and health sciences. Furthermore, the aerial tissues of *I. noli-tangere* are rich in volatile ALA-derived metabolites, such as (Z)-3-hexenol [13]. These short-chain hydrocarbons play key roles in plant defense, studies have shown that fatty acid-derived C6 volatiles are released during pathogen attacks to disrupt microbial membranes, thereby inhibiting bacterial and fungal growth [14–16]. Together, these attributes underscore the potential of *I. noli-tangere* as a natural source of antimicrobial agents.

Despite these promising features, previous studies have primarily focused on the extraction and profiling of fatty acids and volatile organic compounds (VOCs) in *I. noli-tangere*, along with investigations into its geographic differentiation and ecological adaptation [17–19]. Nevertheless, there has been limited experimental validation of its potential applications in food, healthcare, and agriculture. More importantly, the regulatory networks underlying the biosynthesis of ALA and its metabolic volatiles remain poorly understood. Recent advances in plant genomics have enabled comprehensive analyses of biosynthetic pathways, regulatory networks, and biological functions of metabolites in various plant species [20–22]. However, few comprehensive studies have been conducted on *I. noli-tangere*, and the lack of genomic data for this species has hindered understanding of its evolutionary history, phenotypic diversity, and metabolite biosynthetic pathways [23].

To address these gaps, we assembled the first high-quality genome of *I. noli-tangere* using Illumina, PacBio, and Hi-C technologies. Comparative genomics analysis revealed distinctive evolutionary features of its genome, including mechanisms of long terminal repeat retrotransposons (LTR-RTs) elimination that maintain genome size and the significant contribution of whole-genome duplication (WGD) to oil biosynthesis. Integrating multi-omics approaches with experimental validations, we identified *IntbZIP38* as a key transcription factor that positively regulates *IntFAD3*, a critical gene in ALA biosynthesis. Additionally, antifungal assays demonstrated that the high (E)-2-hexenal content in *I. noli-tangere* leaf essential oil effectively inhibits the growth of *Botrytis cinerea*. These findings illuminate the molecular basis of ALA biosynthesis and the antifungal properties of VOCs in *I. noli-tangere*, establishing a robust foundation for its applications in food science, healthcare, and agriculture, particularly as a natural antifungal resource.

## Results

### Genome sequencing and assembly

**Figure 1:**
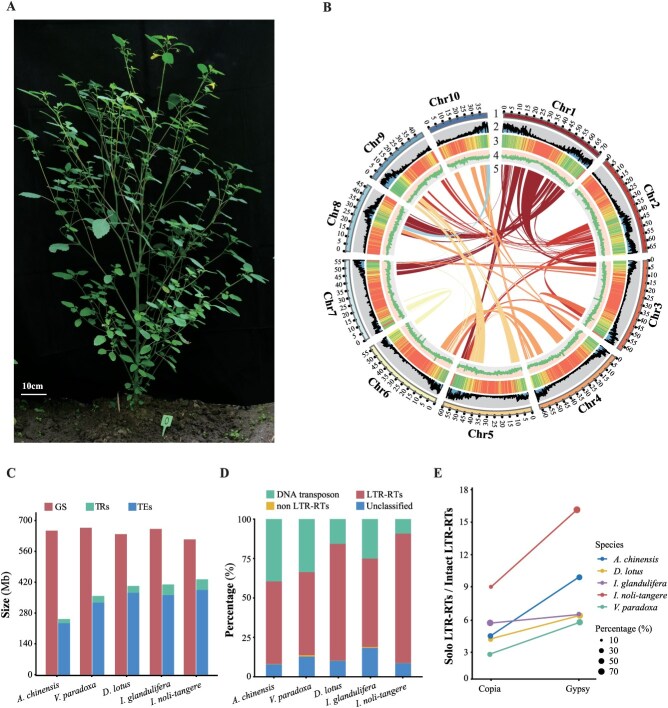
Morphological and genome assembly characteristics of *Impatiens noli-tangere*. (A) Morphological features of *I. noli-tangere* at the flowering stage. Scale bar: 10 cm. (B) Overview of the assembled genome of *I. noli-tangere*. The tracks indicate the distribution of diverse genome features. Bar plots from outside to inside represent: (1) Chromosome length (Mb); (2) Protein-coding gene density; (3) Transposable element (TE) density; (4) GC content; (5) Schematic presentation of major interchromosomal relationships. The sliding window size for GC content, gene, and TE is 100 kb. (C) Comparison of repeat sequence composition in the genomes of *I. noli-tangere* and other Rhododendroales plants, including *Actinidia chinensis*, *Vitellaria paradoxa*, *Diospyros lotus*, and *I. glandulifera*. GS, Genome size; TRs, Tandem repeats; TEs, Transposable elements. (D) Comparison of transposable element composition in the genomes of *I. noli-tangere* and other Rhododendroales plants. (E) Ratio of Solo LTR-RTs to Intact LTR-RTs of Copia and Gypsy elements of LTR-RTs in the genomes of *I. noli-tangere* and other Rhododendroales plants. The size of the circles represents the proportion of Copia or Gypsy elements.

This study employed a combination of second- and third-generation sequencing technologies to sequence the genome of *I. noli-tangere*. A total of 37.96 Gb of Illumina short reads and 42.02 Gb of PacBio single-molecule long reads were obtained (Fig. 1A, Table S1). K-mer analysis (K-mer = 17) of the short reads indicated that the genome size of *I. noli-tangere* is ~677.41 Mb, with a heterozygosity rate of 0.20% and a repeat sequence proportion of 71.22%, highlighting the genome's high complexity (Fig. S1, Table S2).

To enhance the assembly quality, error correction and assembly of the long reads were conducted, resulting in a draft genome of 614.46 Mb with a Scaffold N50 of 59.74 Mb (Table S3). Benchmarking Universal Single-Copy Orthologs (BUSCO) analysis revealed that 97.89% of BUSCO genes were present in the assembled genome, indicating a high level of genomic integrity (Table S4).

Chromosome-level scaffolding was performed by integrating high-throughput chromatin conformation data generated through Hi-C sequencing. A total of 62.70 Gb of raw reads were obtained, representing ~102.04× genome coverage and enabling the ordering and orientation of scaffolds into chromosome-scale assemblies. This resulted in the assembly of 10 chromosomes, with a total length of 566.77 Mb, covering 92.24% of the assembled genome (Fig. 1B, Table S3). The Hi-C interaction matrix exhibited pronounced diagonal signal patterns, confirming the absence of large-scale misassemblies or chromosomal inversions (Fig. S2).

### Genome annotation

#### Protein-coding genes and non-coding RNAs

With the assembled genome as a foundation, gene annotation was performed to reveal the composition and structural features of coding and non-coding elements. Protein-coding genes were annotated to a combination of *ab initio*, homology-based, and transcriptome-assisted methods. A total of 27864 gene models were predicted in the genome of *I. noli-tangere*. The average gene length is 2910.41 bp, with coding sequences, exons, and introns averaging 1209.47, 231.60, and 402.84 bp, respectively (Table S5). Functional annotation of the protein-coding genes revealed that 98.41% (27420) of these genes possess explicit functional annotations in public databases including SwissProt, Pfam, and KEGG (Table S6). In addition to protein-coding genes, 387 microRNAs (miRNAs), 651 small nuclear RNAs (snRNAs), 4670 transfer RNAs (tRNAs), and 26682 ribosomal RNAs (rRNAs) were identified in the genome (Table S3).

#### Repetitive sequences

Given the unexpectedly high proportion of repetitive sequences revealed by initial genome survey, a comprehensive analysis of transposable elements (TEs) was conducted. In total, 432.97 Mb of repetitive sequences were identified in the *I. noli-tangere* genome, with TEs as the predominant component, contributing 384.74 Mb (Fig. 1C). Among these, LTR-RTs comprised the majority, accounting for 82.06% (315.72 Mb) of the total TEs. Notably, Gypsy elements represented 82.22% (259.59 Mb) of the LTR-RTs, while Copia elements constituted 16.68% (52.66 Mb) (Fig. 1D and E).

The composition of repetitive sequences in the *I. noli-tangere* genome mirrors that of four other species in the order Rhododendroales, including *Actinidia chinensis*, *Vitellaria paradoxa*, *Diospyros lotus*, and *I. glandulifera*. In these genomes, LTR-RTs also account for over 50% of TEs (Fig. 1D), and both Gypsy and Copia elements have experienced significant amplification within the timeframe of 0.40 to 1.00 million years ago (Mya) (Fig. S3). Notably, despite the relatively small genome size of *I. noli-tangere* (614.46 Mb), TEs represent the highest proportion, reaching 62.61% (Fig. 1C).

Further analysis of the LTR-RTs across these five plant species revealed that the ratios of solo LTR-RTs to intact LTR-RTs (S/I) for Gypsy and Copia in *I. noli-tangere* were 16.30 and 9.02, respectively, marking the highest values observed among these species (Fig. 1E). In contrast, the S/I ratios for Gypsy in the other species were as follows: *A. chinensis* (9.93), *I. glandulifera* (6.48), *D. lotus* (6.38), and *V. paradoxa* (5.74). For Copia, the S/I ratios were as follows: *I. glandulifera* (5.70), *A. chinensis* (4.54), *D. lotus* (4.25), and *V. paradoxa* (2.87).

### Genome evolution

**Figure 2:**
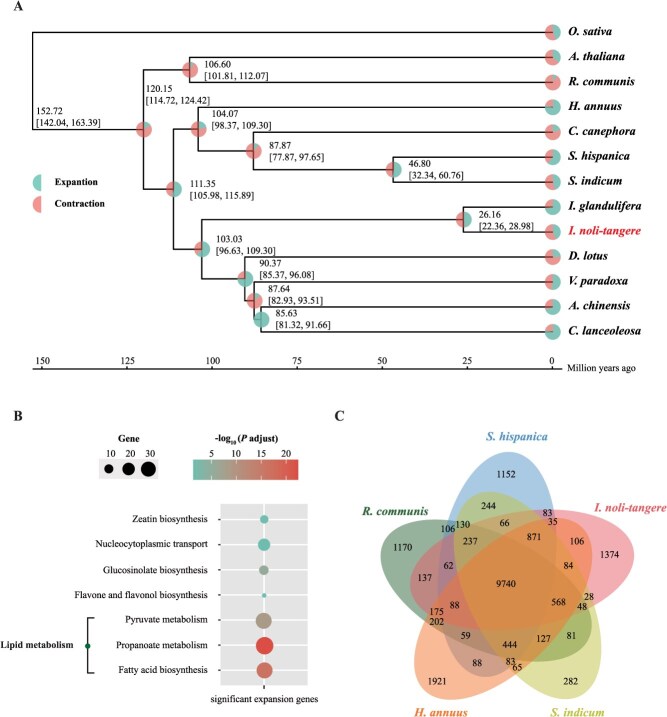
Phylogenetic and gene family analysis of *I. noli-tangere*. (A) Phylogenetic analysis of *I. noli-tangere* and twelve other species. Each node indicates the calculated divergence time and its 95% confidence interval. The pie chart shows the proportions of gene families that underwent expansion or contraction. (B) KEGG enrichment analysis of expanded gene families in *I. noli-tangere*. The figure displays all KEGG enrichment entries with *P* < 0.05. The size of the circles indicates the number of genes in each KEGG entry, while the color represents significance. (C) Venn diagram of gene families among *I. noli-tangere*, *Ricinus communis*, *Helianthus annuus*, *Sesamum indicum*, and *Salvia hispanica*.

To further understand the evolutionary context of the annotated genome, phylogenetic and gene family analyses were performed. Phylogenetic reconstruction and divergence time estimation were conducted based on 396 single copy genes across 13 plant genomes. *Oryza sativa* was utilized as the outgroup, alongside six species from the order Rhododendroales, four from Asterales, and two from Brassicales. The genomic evolutionary analysis indicated that the family Balsaminaceae diverged from Ebenaceae ~103.03 Mya. Moreover, *I. noli-tangere* and *I. glandulifera* formed a monophyletic clade, with an estimated divergence of around 26.16 Mya (Fig. 2A).

Analysis of the 31581 orthologous gene families revealed that 54 gene families in *I. noli-tangere* underwent significant expansion (*P* < 0.05), while 135 gene families exhibited significant contraction (*P* < 0.05) (Fig. 2A). The expanded gene families were predominantly enriched in pathways associated with fatty acid biosynthesis, flavonoid biosynthesis, and zeaxanthin biosynthesis, thereby establishing a genetic foundation for the accumulation of these metabolites in *I. noli-tangere* (Fig. 2B). Furthermore, in comparison to oil crops, such as *Ricinus communis*, *Helianthus annuus*, *Sesamum indicum*, and *Salvia hispanica*, *I. noli-tangere* possessed only 1374 unique gene families, significantly fewer than the 9740 shared gene families among these plants (Fig. 2C). The shared gene families were notably enriched in carbohydrate metabolism and lipid metabolism pathways (*P* < 0.05) (Fig. S4), further indicating the potential richness of fatty acid biosynthesis and metabolic-related genes in *I. noli-tangere*.

### Gene duplication analysis

#### Identification of WGD events

**Figure 3:**
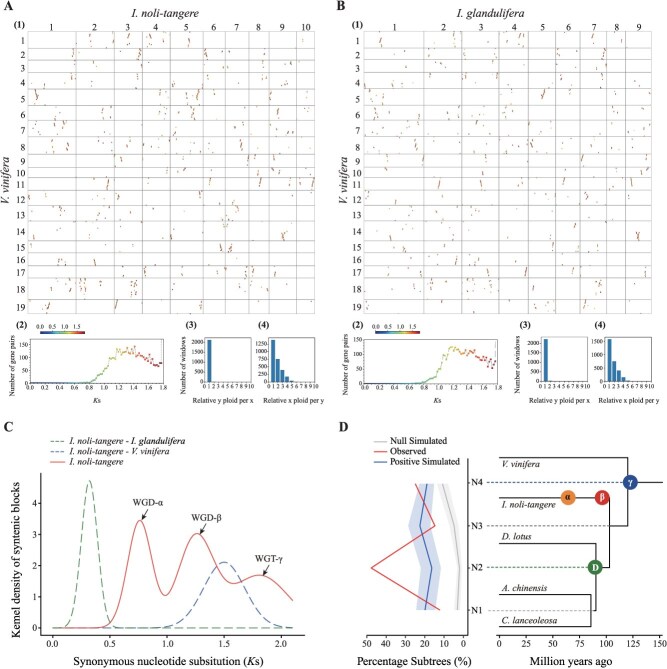
Whole-genome duplication (WGD) analysis of *I. noli-tangere*. (A, B) Synonymous substitution (*K*s)-colored dot plots of orthologous synteny after applying an Orthology Index cutoff of 0.6, illustrating a clear one-to-four orthology between *Vitis vinifera* and each of *I. noli-tangere* and *I. glandulifera*. (1) Dot plots color-coded by *K*s, where each dot represents an orthologous gene pair between the two genomes. (2) Corresponding *K*s histograms using the same color scale as the dot plots. (3–4) Orthologous synteny depth across 50-gene windows. (C) *K*s distributions of paralogous genes in *I. noli-tangere*, and of orthologous genes between *I. noli-tangere* and each of *I. glandulifera*, and *V. vinifera*. (D) MultiAxon Paleopolyploidy Search **(**MAPS) results from observed data, null and positive simulations on the associated phylogeny. Percentage of subtrees that contain a gene duplication shared by descendant species at each node, results from observed data, 100 resampled sets of null simulations, and positive simulations.

Considering the importance of gene duplication in genome evolution, WGD events in *I. noli-tangere* were identified and characterized. Orthologous synteny analysis based on the Orthology Index revealed a 4:1 syntenic block ratio between *I. noli-tangere* and *Vitis vinifera*, which experienced only the ancestral core-eudicot whole-genome triplication (WGT-γ) (Fig. 3A). This ratio matches that between *I. glandulifera* and *V. vinifera*, suggesting that members of the genus *Impatiens* underwent two lineage-specific WGDs after WGT-γ (Fig. 3B). Further comparison with *Camellia lanceoleosa*, which experienced WGT-γ plus one additional WGD, revealed a syntenic block ratio of 4:2, supporting one more WGD in *I. noli-tangere* (Fig. S5).

Analysis of synonymous substitution rates (*K*s) within the *I. noli-tangere* genome provided complementary evidence. The *K*s distribution of paralogous genes displayed three distinct peaks (Fig. 3C). The oldest peak (*K*s ≈ 1.81) corresponds to WGT-γ, whereas the youngest peak (*K*s ≈ 0.76) and the intermediate peak (*K*s ≈ 1.25) represent two more recent WGD events. This interpretation is reinforced by the *K*s peak of orthologous gene pairs between *I. noli-tangere* and *V. vinifera* (*K*s ≈ 1.50) (Fig. 3C). This value is lower than the ancestral WGT-γ peak (*K*s ≈ 1.81) but higher than the older lineage-specific WGD peak (*K*s ≈ 1.25). Based on this, the two more recent WGD events are dated to ~60.88 and 100.13 Mya, respectively (Table S7). *I. glandulifera* and *I. noli-tangere* share extensive 1:1 synteny and a *K*s peak of 0.25 for orthologous genes, indicating they diverged after the two shared WGDs (Fig. 3C, Fig. S5). By contrast, the *K*s peak for *C. lanceoleosa* paralogs (*K*s ≈ 0.47) is lower than that observed for orthologs between *C. lanceoleosa* and *I. noli-tangere*, suggesting that the recent WGD in *I. noli-tangere* is not shared with *C. lanceoleosa* (Fig. S5).

To further verify the phylogenetic positions of these WGD events, we applied the MultiAxon Paleopolyploidy Search (MAPS) algorithm using *O. sativa* as the outgroup and *V. vinifera* as a representative core eudicot. Across the Ericales species examined (*A. chinensis*, *C. lanceoleosa*, *D. lotus*, and *I. noli-tangere*), significantly elevated proportions of gene-duplicated subtrees were detected at nodes N2 and N4 (*P* < 0.01), even under positive simulations assuming WGD at each node (Fig. 3D). These findings corroborate the *K*s-based inference that the two WGD events in *I. noli-tangere* are lineage-specific and not shared with other closely related families such as Actinidiaceae, Ebenaceae, or Theaceae.

#### Duplication and functional analysis of oil biosynthetic genes

**Figure 4:**
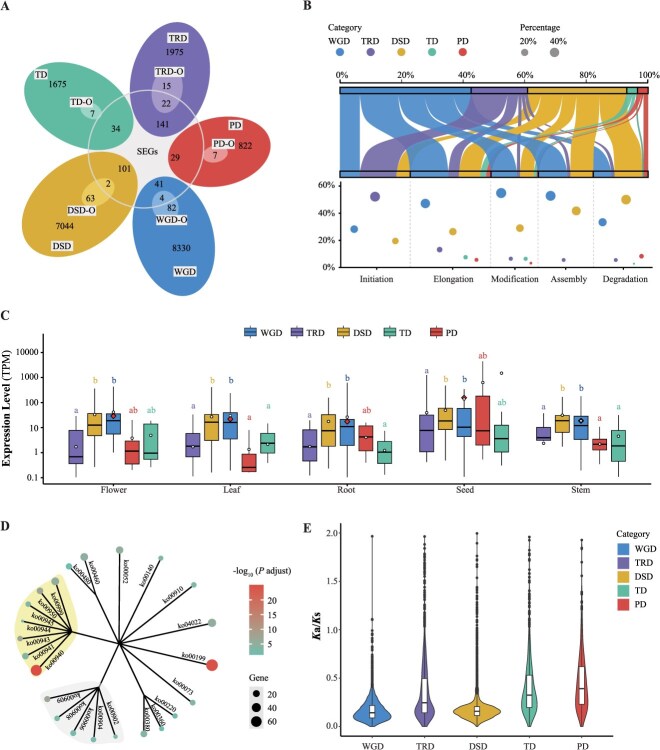
Characterization of oil biosynthetic genes and their duplication modes in *I. noli-tangere*. (A) Venn diagram of significantly expanded genes and various types of duplicated genes (WGD, TD, PD, TRD, and DSD). TD-O, tandem duplicates in oil biosynthesis; PD-O, proximal duplicates in oil biosynthesis; DSD-O, dispersed duplicates in oil biosynthesis; TRD-O, transposed duplicates in oil biosynthesis; WGD-O, whole-genome duplicates in oil biosynthesis; SEGs, significantly expanded genes. (B) Proportions of oil biosynthetic genes derived from different duplication modes across various biosynthetic stages. The upper panel displays a mulberry plot showing the distribution of gene duplication types across five stages of oil biosynthesis, including initiation, elongation, modification, assembly, and degradation. The lower panel presents a bubble chart quantifying the relative contribution of each duplication type within each biosynthetic stage. Larger bubbles represent higher proportions of genes derived from the corresponding duplication mode. (C) Expression levels of oil biosynthetic genes derived from different duplication modes across various tissues. Different letters indicate statistically significant differences (*P* < 0.05). Red diamonds represent the average expression levels of oil biosynthetic genes in each tissue, while circles indicate the average expression levels of genes from each duplication type. (D) KEGG enrichment analysis of TD genes. The figure presents all KEGG entries with *P* < 0.05. The size of the circles indicates the number of genes in each KEGG entry, while the color represents significance. Yellow branches indicate pathways involved in terpene and polyketide biosynthesis, while gray branches represent pathways related to the biosynthesis of other secondary metabolites. (E) Distribution of *K*a (nonsynonymous substitution)/*K*s ratios among different pairs of duplicated genes. Points outside the box in the boxplot represent outliers, the central line indicates the median, and the box represents the interquartile range.

In the evolutionary history of *I. noli-tangere*, genes involved in oil biosynthesis, such as fatty acid biosynthesis, have undergone significant expansion (Fig. 2B, Fig. S6). A total of 202 oil biosynthetic genes were identified as being derived from gene duplication, with WGD accounting for the largest proportion (86 genes, 42.57%) (Fig. 4A and B, Table S8). Functional categorization indicated that WGD-derived genes were predominantly enriched in the elongation (47.17%), modification (54.84%), and assembly (52.78%) stages of oil biosynthesis. In contrast, although transposed duplication (TRD) accounted for only 18.32% of the duplicated oil biosynthetic genes, it contributed most of the genes involved in the initiation stage (52.17%) (Fig. 4B).

Transcriptome analysis showed that oil biosynthetic genes displayed the highest average expression in seeds, reaching a mean of 155.95 transcripts per million (TPM), underscoring their essential role in seed oil accumulation (Fig. 4C, Table S9). Across all tissues, WGD-derived genes exhibited significantly higher expression levels than TRD-derived genes, suggesting that WGD plays a dominant role in the transcriptional regulation of oil biosynthesis.

Tandem duplication (TD) and proximal duplication (PD) each contributed only 7 genes (3.47%) to the oil biosynthetic genes. Notably, TD-derived genes were significantly enriched (*P* < 0.05) in the biosynthetic pathways of cutin, suberin, wax, phenylpropanoids, sesquiterpenes, and triterpenes (Fig. 4D). Similarly, PD-derived genes were significantly enriched in monoterpenoid and other secondary metabolite biosynthesis (Fig. S7). Further analysis of *K*a (nonsynonymous substitution)/*K*s ratios showed that TD- and PD-derived genes had higher average values (0.47 and 0.53, respectively) compared with other duplication types, suggesting that they may have undergone stronger adaptive evolution (Fig. 4E).

### Detection of ALA content and identification of key genes in its biosynthesis

**Figure 5:**
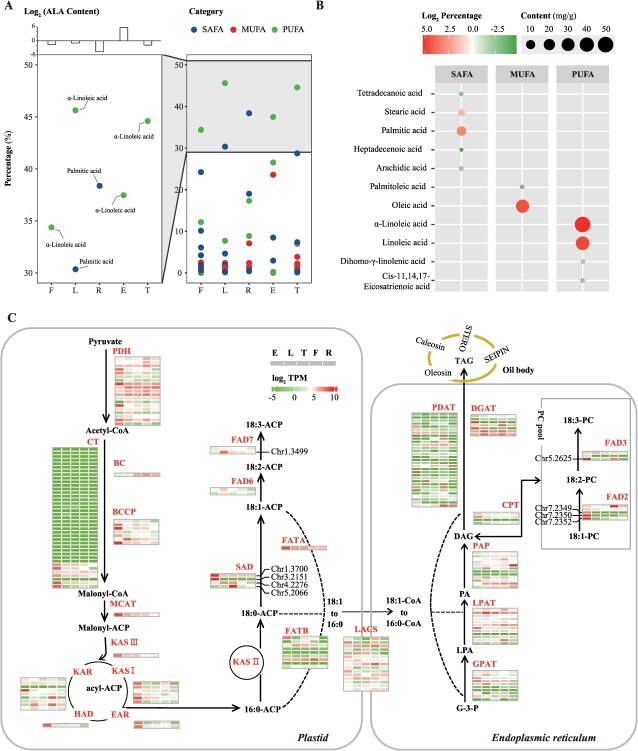
Tissue-specific fatty acid composition and gene expression in oil biosynthesis of *I. noli-tangere*. (A) Comparative analysis of fatty acid composition in the root, stem, leaf, and flower of *I. noli-tangere*. E, seed; L, leaf; T, stem; F, flower; R, root; SAFA, saturated fatty acids; MUFA, monounsaturated fatty acids; PUFA, polyunsaturated fatty acid. (B) Composition of fatty acid compounds in seeds. The size of the circles indicates the content of each fatty acid compound, while the color represents the proportion of that compound. (C) Expression levels of genes in the oil biosynthetic pathway. Relative expression levels are represented as log_2_ (fold change in transcription levels). PDH, pyruvate dehydrogenase; CT, carboxyltransferase; BC, biotin carboxylase; BCCP, biotin carboxyl carrier protein; MCAT, malonyl-CoA: ACP transacylase; KAS, ketoacyl-ACP synthase; KAR, beta-ketoacyl-ACP reductase; HAD, beta-hydroxyoctanoyl-ACP-dehydrase; EAR, enoyl-ACP reductase; SAD, stearoyl-ACP desaturase; FATA, acyl-ACP thioesterase A; FATB, acyl-ACP thioesterase B; LACS, long-chain acyl-CoA synthetase; DGAT, diacylglycerol acyltransferase; PDAT, phospholipid diacylglycerol acyltransferase; PAP, phosphatidic acid phosphatase; LPAT, lysophosphatidic acid acyltransferase; GPAT, glycerol-3-phosphate acyltransferase; CPT, cholinephosphotransferase; FAD2/6, acyl-lipid omega-6 desaturase; FAD3/7, acyl-lipid omega-3 desaturase; PC, phosphatidyl choline.

WGD events in the *I. noli-tangere* genome played a key role in driving functional innovation, particularly in oil biosynthesis. This observation prompted further investigation into the biosynthesis and regulation of ALA, a critical polyunsaturated fatty acid (PUFA) with diverse biological functions. Fatty acid analysis revealed that ALA constitutes over 30% of the fatty acid extracts from seeds, leaves, stems, and flowers of *I. noli-tangere* (Fig. 5A). The highest ALA content was found in the seeds, reaching 50.45 mg/g, followed by leaves (0.52 mg/g), flowers (0.32 mg/g), and stems (0.28 mg/g), with the lowest content in roots (0.04 mg/g) (Fig. 5A and B). Moreover, the alignment rates of transcriptomic data with the reference genome exceeded 85% across all tissues, indicating a high compatibility between the transcriptome and reference genome, which is suitable for analyzing the expression patterns of genes involved in ALA biosynthesis (Table S9).

In ALA biosynthesis, the ω-3 fatty acid desaturase gene encodes an enzyme that introduces an additional double bond into the carbon chain of fatty acids, converting linoleic acid (LA, 18:2) into ALA (18:3) (Fig. 5C). Five ω-3 FADs were identified, among which *IntFAD7* (*Chr1.3499*) and *IntFAD3* (*Chr5.2625*), both derived from WGD, were specifically expressed in leaves and seeds, respectively (Fig. 5C, Figs S8 and S9). Notably, in the seeds, the expression level of *IntFAD3* reached 8405.70 TPM, significantly higher than in other tissues (Fig. 5C). To validate its function, *IntFAD3* was fused into the PBI121 vector and overexpressed in the model plant *Nicotiana tabacum*. Compared to empty vector transgenic plants, the ALA content in the leaves of *N. tabacum* overexpressing *IntFAD3* significantly increased by 28.72% (*P* < 0.05) (Fig. S10).

**Figure 6:**
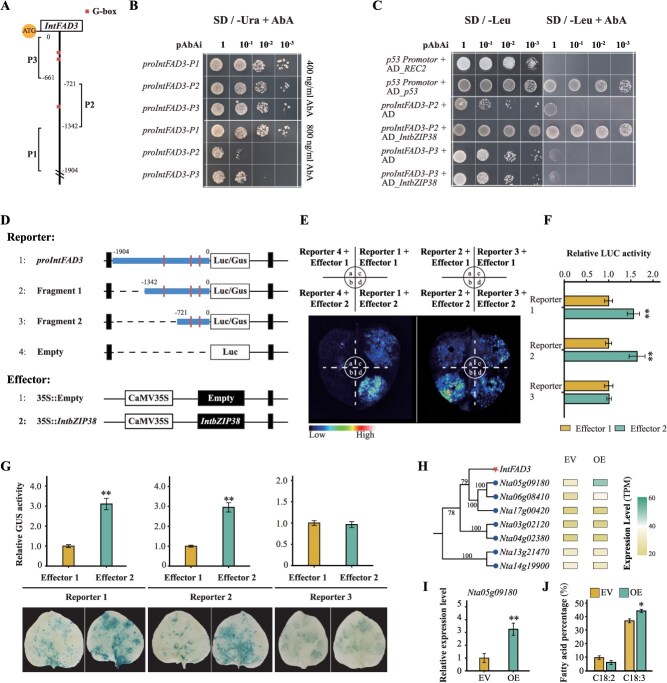
Binding and transcriptional regulation of *IntbZIP38* to the *IntFAD3* promoter. (A) Distribution of G-box elements in the *IntFAD3* promoter. (B) Growth of yeast strains carrying recombinant pAbAi plasmids with *proIntFAD3-P1*, *proIntFAD3-P2*, and *proIntFAD3-P3* under different AbA concentrations. SD, synthetic dropout medium; URA, uracil. (C) Binding analysis of *IntbZIP38* with *proIntFAD3-P2* and *proIntFAD3-P3*. Leu, leucine. (D) Schematic representation of reporter and effector plasmids used in LUC and GUS assays. (E, F) Effect of *IntbZIP38* on the activation of the LUC gene by the *proIntFAD3*. Error bars represent the mean ± SD of three biological replicates. Asterisks indicate significant differences compared to the control (***P < 0.001, **P < 0.01, *P < 0.05; same for subsequent figures). (G) Effect of *IntbZIP38* on the activation of the GUS gene by *proIntFAD*. (H, I) Comparative expression analysis of the *N. tabacum* homolog closely related to *IntFAD3* in empty vector (EV) and *IntbZIP38*-overexpressing line by RNA-seq and RT-qPCR. (J) Proportional analysis of LA and ALA in the leaf fatty acid profile of *IntbZIP38*-expressing *N. tabacum*.

*Pearson* correlation analysis revealed a significant positive correlation between the expression levels of the *IntbZIP38* (*Chr10.38*) and the *IntFAD3* (*P* < 0.001) (Fig. S11). Promoter analysis showed that the promoter region of the *IntFAD3* gene contains three G-box elements specifically bound by *bZIP* transcription factors (Fig. 6A). Subsequent Y1H assays demonstrated that *IntbZIP38* could bind to the *proIntFAD3-P2* promoter containing the G-box elements, promoting the growth of *S. cerevisiae* on SD/−Leu medium supplemented with 800 ng/ml AbA (Fig. 6B and C). However, *proIntFAD3-P1* exhibited high self-activation levels that could not be effectively suppressed, making it unsuitable for Y1H assays. In addition, LUC assays further indicated that *IntbZIP38* significantly enhanced the expression of the luciferase reporter driven by the *proIntFAD3* promoter (*P* < 0.01) (Fig. 6D–F). Conversely, deletion of the *proIntFAD3-P2* fragment abolished this enhancement, as indicated by the lack of significant changes in LUC activity (*P* > 0.05). Consistent with these findings, GUS staining and quantitative analysis further confirmed the regulatory effect of *IntbZIP38* on *IntFAD3* expression (Fig. 6G and H). Furthermore, expression analysis in transgenic *N. tabacum* lines revealed that the expression of *Nta05g09180*, the closest homolog of *IntFAD3* in tobacco, was significantly upregulated by *IntbZIP38* overexpression (Fig. 6H and I). Fatty acid composition analysis showed that the relative content of ALA (C18:3) was significantly increased (*P* < 0.05), whereas the proportion of LA (C18:2) exhibited a decreasing trend in the leaves of *IntbZIP38*-overexpressing line (Fig. 6J). These results suggest that *IntbZIP38* positively regulates *IntFAD3* expression and promotes the accumulation of ALA in transgenic tobacco leaves.

### Identification of volatile compounds in aerial tissues and their effects on the growth of *B. cinerea*

Although ALA was predominantly enriched in seeds, its quantifiable levels in leaves, stems, and flowers implied potential roles beyond storage (Fig. 5A and B). To investigate whether ALA in these aerial tissues serves as a precursor for antifungal volatile compounds, volatile profiles of these tissues were analyzed using solid-phase microextraction combined with gas chromatography–mass spectrometry (SPME-GC–MS). Aliphatic compounds and terpenes collectively accounted for over 80% of the volatiles across all three tissue types (Fig. S12). Aliphatic compounds were predominant in leaves (63.26%) and flowers (52.93%), while stems exhibited a terpene-rich profile, with terpenes comprising 53.46% of total volatiles and aliphatic compounds 28.35% (Fig. S12). Among individual compounds, (E)-2-hexenal was the most abundant aliphatic compound in leaves (52.15%), whereas linalool was the dominant terpene in stems (27.11%) (Fig. S13).

**Figure 7:**
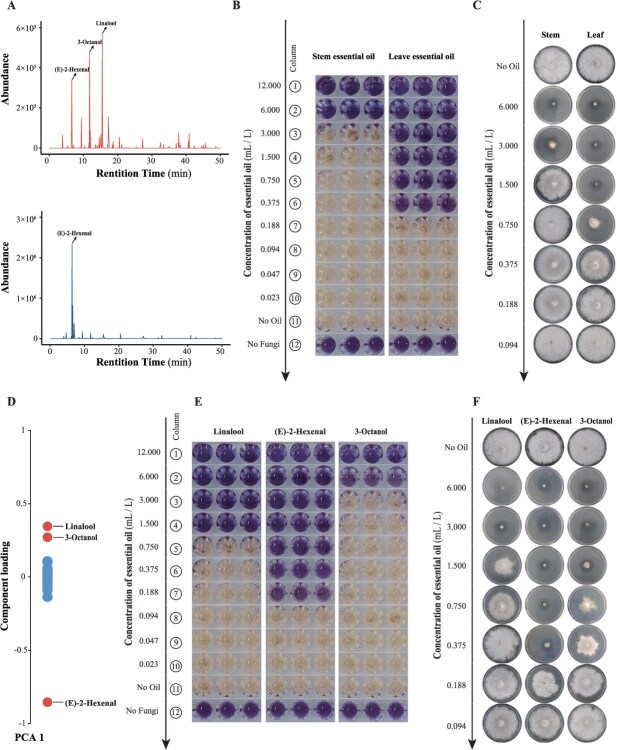
Inhibitory effects of essential oils from stems and leaves and their major compounds on *Botrytis cinerea*. (A) GC–MS peaks of volatile components in essential oils from stems and leaves. (B) Minimum inhibitory concentration (MIC) of essential oils from stems and leaves of *I. noli-tangere* against *B. cinerea*. The threshold for inhibition is determined by resazurin reduction test, where color changes from blue (oxidized state) to pink or colorless (reduced state) indicate fungal growth activity. MIC is defined as the lowest concentration of essential oil that prevents color change. (C) Effect of essential oils from stems and leaves on the growth of *B. cinerea* mycelium. (D) Analysis of the main components in the essential oils from stems and leaves. (E) MIC of (E)-2-hexenal, 3-octanol, and linalool against *B. cinerea*. (F) Effects of (E)-2-hexenal, 3-octanol, and linalool on the growth of *B. cinerea* mycelium.

To evaluate the antifungal activity of these major volatile compounds, essential oils were extracted from leaves and stems and subjected to GC–MS analysis. The composition of the essential oils closely matched that of the native volatiles, confirming their suitability for bioactivity assays (Fig. 7A). Cell viability assays against *B. cinerea* revealed that the leaf essential oil exhibited significantly stronger antifungal activity. The minimum inhibitory concentration (MIC) of the leaf essential oil was 0.375 ml/l, compared to 6.000 ml/l for the stem essential oil (Fig. 7B). At a concentration of 0.750 ml/l, leaf essential oil inhibited hyphal growth by 69.44%, whereas the stem essential oil showed no inhibitory effect (Fig. 7C, Fig. S14).

Analysis of the main components in the essential oils indicated that the primary differing components were (E)-2-hexenal, linalool, and 3-octanol (Fig. 7D). Among these, (E)-2-hexenal showed the strongest antifungal activity, with an MIC of 0.188 ml/l and an 83.67% inhibition of hyphal growth at 0.375 ml/l (Fig. 7E and F, Fig. S14). In contrast, at the same concentration (0.375 ml/l), linalool and 3-octanol inhibited hyphal growth by only 12.23% and 38.65%, respectively. Their MICs were much higher, at 1.500 and 6.000 ml/l. Compared to (E)-2-hexenal, the other ALA-derived compound (Z)-3-hexenol also inhibited *B. cinerea*, achieving 80.33% hyphal growth inhibition, but only at a relatively high MIC of 3.000 ml/l (Figs S14 and S15).

### Distribution of ALA metabolic volatiles in aerial tissues and identification of key genes in its biosynthesis

**Figure 8:**
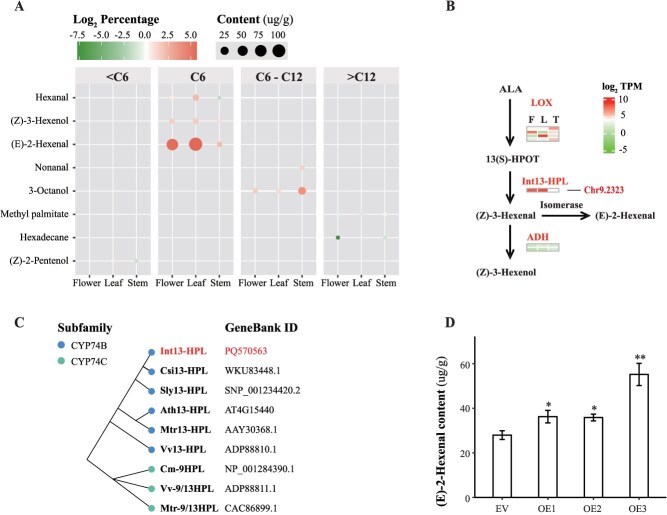
Detection of ALA metabolic volatiles in different tissues of *I. noli-tangere* and identification of key genes in their biosynthesis. (A) Composition of aliphatic compounds in the volatiles from the stem, leaf, and flower. (B) Expression levels of genes in the ALA metabolic volatiles biosynthesis. F, flower; L, leaf; T, stem; LOX, lipoxygenase; HPL, hydroperoxide lyase; ADH, alcohol dehydrogenase class-P. (C) Phylogenetic tree of *13-HPL* gene in *I. noli-tangere*, and other representative plant species *based* on the neighbor-joining algorithm. Csi, *C. sinensis*; Sly, *Solanum lycopersicum*; Ath, *Arabidopsis thaliana*; Mtr, *Medicago truncatula*; Vv, *V. vinifera*; Cm, *Cucumis melo*. (D) Content of (E)-2-hexenal in the leaves of three transgenic *N. tabacum* lines overexpressing *Int13-HPL*.

Due to the notable difference in antifungal activity between ALA-derived volatiles, we examined their tissue-specific distribution and associated biosynthetic pathway. Volatile profiling demonstrated consistent predominance of (E)-2-hexenal over (Z)-3-hexenol across all examined tissues. Leaves contained the highest (E)-2-hexenal concentration (108.44 μg/g), which was 17.78-fold greater than that of (Z)-3-hexenol (6.10 μg/g). Similar fold differences were observed in flowers (16.07-fold) and stems (2.52-fold) (Fig. 8A).

In the ALA-derived volatile biosynthetic pathway, HPL catalyzes the formation of (Z)-3-hexenal and its isomer (E)-2-hexenal. These compounds serve as substrates for ADH-mediated reduction to the corresponding alcohols (Fig. 8B). Although all *IntADH* genes exhibited basal expression across tissues (TPM < 10), *Int13-HPL* showed strong tissue-specific expression, peaking in leaves (TPM = 386.15) and flowers (TPM = 323.18), which coincided with high (E)-2-hexenal accumulation. Conversely, its diminished expression in stems (TPM = 31.25) corresponds to the lowest (E)-2-hexenal concentration. These results indicate that *Int13-HPL* plays a critical role in (E)-2-hexenal biosynthesis.

Phylogenetic analysis clustered *Int13-HPL*, a member of the CYP74B subfamily, closely with *Csi13-HPL* from *C. sinensis* (Fig. 8C). To verify its function, *Int13-HPL* was overexpressed in *N. tabacum*. Compared to the control line transformed with the empty vector PBI121, leaves of the overexpression line OE3 exhibited a significant increase in (E)-2-hexenal content by 91.43% (*P* < 0.01) (Fig. 8D), confirming the critical role of *Int13-HPL* in (E)-2-hexenal biosynthesis.

## Discussion

The abundant ALA in *I. noli-tangere* is not only an essential fatty acid required in human diets but also a critical metabolite for maintaining plant physiological functions [6, 24]. This study presented a high-quality chromosome-level genome of *I. noli-tangere* using Illumina, PacBio, and Hi-C sequencing technologies. By integrative multi-omics analyses with functional validation, the synthetic and metabolic mechanisms of ALA were systematically elucidated. Additionally, the antifungal activity of (E)-2-hexenal, an ALA-derived volatile compound abundant in *I. noli-tangere* leaf essential oil, was assessed. These findings provide new insights into the molecular mechanisms underlying ALA biosynthesis and metabolism in *I. noli-tangere*, while also offering valuable references for the potential agricultural and health applications of this species.

The genome size of *I. noli-tangere* is 614.46 Mb, with a chromosome attachment rate of 92.24%. Despite the genus *Impatiens* comprising over 1000 species, genomic studies remain limited. The genome presented here achieves 97.89% BUSCO completeness, significantly exceeding the 80.61% completeness of the *I. capensis* genome [23], establishing it as a valuable resource for *Impatiens* research. Genome annotation revealed that TEs account for 62.61% of the genome, a proportion higher than analyzed Ericales species. TEs are often pivotal in genome expansion, as observed in giga-genome of *Paeonia ostia* [22]. Despite the high TE content, the *I. noli-tangere* genome remains compact, likely due to efficient LTR-RTs removal mechanisms. This is evidenced by the significantly higher ratio of solo LTR-RTs to intact LTR-RTs compared to other plants. Such mechanism is essential for maintaining genome compactness in the face of extensive repetitive sequences and has likely played a crucial role in the evolutionary stability of genome size.

The assembled genome also provides valuable insights into the evolutionary history of *I. noli-tangere*, revealing two WGD events dated to ~100.13 and 60.88 Mya. Notably, the more recent WGD event coincides with the Cretaceous–Paleogene (K–Pg) boundary, a period marked by the extinction of 30% to 60% of plant species [25, 26]. This temporal association suggests that WGD may have served as an adaptive mechanism, enhancing the survival of *I. noli-tangere* amid drastic environmental changes. Similar WGD events in *Protea cynaroides*, *Zea mays*, and *Solanum tuberosum* further support this adaptive role [27, 28]. Following these WGD events, *I. noli-tangere* exhibits significant expansion of gene families enriched in fatty acid biosynthesis. Comparable expansions of oil biosynthesis-related gene families have also been observed in major oil crops such as *S. indicum*, *Olea europaea*, and *Glycine max* [29–31]. The widespread occurrence of this feature among oil-producing species suggests that WGD is an important evolutionary driver of oil accumulation traits. Further supporting this, our detailed analysis demonstrates that WGD-derived genes constitute the largest proportion of oil biosynthetic genes in *I. noli-tangere*. This trend is consistent across the same oil crops noted above. Specifically, WGD-derived genes account for 58.06%, 79.13%, and 87.82% of oil biosynthetic genes in *S. indicum, O. europaea,* and *G. max*, respectively [32]. This recurrent pattern across phylogenetically distinct oil crops indicates that WGD-derived genes provide an essential genetic basis for enhanced oil accumulation traits.

However, the diversity and specific composition of fatty acids in seed oils may not be determined solely by gene duplication resulting from WGD. ω3-FADs catalyze the desaturation of LA to produce ALA, and *I. noli-tangere* harbors five ω3-FAD genes, four of which are WGD-derived and exhibit higher expression levels. This appears to correspond with its relatively high ALA content, which exceeds 30% of seed oil. Nevertheless, this apparent correlation does not hold consistently across oil crops. For example, *G. max* contains eight ω3-FAD genes, all WGD-derived, yet accumulates lower levels of ALA [33]. In contrast, *Salvia hispanica* has only four ω3-FAD genes (two WGD-derived) but exhibits the highest ALA content among the three [34]. These comparisons indicate that ALA accumulation is not simply determined by the number of WGD-derived ω3-FAD genes, and that the fatty acid composition is more likely regulated by additional mechanisms. Indeed, recent studies have shown that the catalytic efficiency of ω3-FADs is affected by the geometry of transmembrane pores, and that ALA accumulation is sensitive to environmental temperature [35]. These findings underscore that fatty acid composition is shaped not only by gene copy number and duplication origin but also by enzyme structure and multilayered regulation at both the transcriptional and post-transcriptional levels.

Building on the understanding that fatty acid composition is regulated by multilayered mechanisms, our findings in *I. noli-tangere* delineate a transcriptional regulatory module controlling ALA biosynthesis. Specifically, the seeds of *I. noli-tangere* contain the highest ALA content (50.45 mg/g), emphasizing their nutritional and healthcare value. Transcriptomic analyses revealed that *IntFAD3*, a WGD-derived gene, exhibits seed-specific and high-level expression, suggesting its involvement in ALA accumulation. Heterologous overexpression of *IntFAD3* in *N. tabacum* led to a 28.72% increase in ALA content in transgenic leaves (*P* < 0.05), supporting its functional role in ALA biosynthesis. Homologs of *IntFAD3* have also been functionally characterized in other ALA-rich species such as *P. suffruticosa*, *S. hispanica*, and *Perilla frutescens* [36–38]. To further explore the regulatory mechanisms underlying species-specific differences in ALA biosynthesis, we investigated the transcriptional regulation of *IntFAD3*. Y1H, LUC, and GUS staining assays identified *IntbZIP38*, a member of the bZIP-A subfamily, positively regulates ALA biosynthesis through the transcriptional activation of *IntFAD3*. Heterologous overexpression of *IntbZIP38* in *N. tabacum* significantly induced the expression of the *IntFAD3* homolog *Nta05g09180* and elevated ALA content in transgenic plants (*P* < 0.05). Collectively, these findings indicate that *IntbZIP38* acts as a positive regulator of ALA biosynthesis by transcriptionally activating *IntFAD3*. Interestingly, bZIP-A subfamily members have been implicated in the regulation of PUFA biosynthesis across diverse plant species. For instance, in *C. sativa*, the bZIP-A member *CsbZIP-A12* functions as a nuclear-localized transcriptional activator that directly binds to the promoters of *CsFAD3–3* and *CsSAD2–3*, thereby promoting the accumulation of unsaturated fatty acids in seeds [39]. In addition, hormone-related regulatory mechanisms may also contribute to PUFA biosynthesis. In *P. ostii*, abscisic acid treatment significantly increased PUFA content and upregulated the expression of *PoABI5*. In *Arabidopsis thaliana*, overexpression of *PoABI5* activated multiple lipid metabolism-related genes, including *AtFAD2/3*, *AtLEC1*, *AtWRI1*, *AtFUS3*, and *AtABI3*, ultimately enhancing PUFA accumulation [40]. Whether *I. noli-tangere* employs a comparable hormone- or complex-mediated regulatory mechanism remains to be determined and warrants in-depth functional dissection, such as elucidating upstream signaling triggers or protein interaction networks.

ALA is also a precursor for certain C6 VOCs, which function as natural plant-produced ‘fungicides’ and ‘insecticides’ by activating defense signaling in neighboring plants and exerting direct toxicity against pathogens and pests [14]. SPME-GC–MS analysis revealed that (E)-2-hexenal is the predominant volatile in *I. noli-tangere* leaves, comprising 52.15% of the total volatile composition. This compound strongly inhibits *B. cinerea*, with a MIC of 0.188 ml/l. In addition to *B. cinerea*, (E)-2-hexenal has also been reported to inhibit other phytopathogens, including *Fusarium graminearum* and *Penicillium cyclopium* [41, 42]. This study further demonstrated a dose-dependent inhibitory effect of (E)-2-hexenal on *B. cinerea*, providing quantitative evidence for its potential use in biocontrol. Mechanistically, (E)-2-hexenal is believed to disrupt fungal cell membranes and induce the accumulation of reactive oxygen species, ultimately leading to cell death [43–45]. This dual mechanism enhances antifungal efficacy while reducing the likelihood of resistance development, supporting its potential as a sustainable ‘green fungicide’. Additionally, short-chain aldehydes such as (E)-2-hexenal have been shown to be rapidly emitted by *C. sinensis* in response to biotic stress, where they inhibit spore germination and activate systemic resistance in neighboring plants via jasmonic acid signaling pathway [14]. The marked accumulation of (E)-2-hexenal in *I. noli-tangere* leaf therefore highlights its promising potential for the development of plant-derived antifungal agents.

Notably, significant geographic variation was observed in the VOC composition of *I. noli-tangere* populations. While populations from Harbin, China, and Józefów near Biłgoraj, Poland, primarily emit C6 volatiles, their dominant constituents differ [13]. Plants from Harbin are enriched in the more bioactive (E)-2-hexenal, whereas those from Józefów predominantly emit (Z)-3-hexenol, which exhibits considerably weaker antifungal activity against *B. cinerea* (MIC = 3 ml/l). This regional disparity underscores the Harbin population as a valuable natural source of potent antifungal volatiles. To explore the genetic basis underlying this variation, functional analyses identified *Int13-HPL* as a key gene involved in (E)-2-hexenal biosynthesis. Interestingly, similar biosynthetic mechanisms have been reported in other species. For example, genomic analyses in *Cucumis sativus* revealed that the expansion of *LOX* family genes and tandem duplication of *HPL* genes drive the production of (E, Z)-2,6-nonadienal, a fatty acid-derived VOC that contributes to cucumber's green aroma and exhibits antifungal activity against *F. verticillioides* [46]. The conservation of *HPL*-mediated VOC biosynthesis across divergent species suggests an evolutionarily conserved defense strategy against fungal pathogens. These findings collectively advance our understanding of VOC-mediated plant defense and provide valuable insights for the development of resistance-improvement strategies and sustainable biocontrol solutions.

In summary, this study provides a comprehensive analysis of the *I. noli-tangere* genome, highlighting the role of LTR-RTs elimination and WGD in genome evolution. It identifies *IntbZIP38* as a key regulator of ALA biosynthesis and demonstrates the antifungal potential of ALA-derived volatiles, particularly (E)-2-hexenal. These findings provide critical genomic insights and highlight *I. noli-tangere*'s potential for advancing sustainable agriculture and bioactive compound production. Future research should leverage gene-editing technologies to validate the functions of key genes, investigate upstream regulatory pathways, and explore the antimicrobial spectrum of *I. noli-tangere* VOCs, maximizing their ecological and economic value.

## Materials and methods

### Plant materials

In April 2022, seedlings of *I. noli-tangere* were introduced from the Maoershan Experimental Forest Farm of Northeast Forestry University to the Northeast Forestry University nursery. After 2 months of cultivation, tender leaves from the same plant were collected for whole-genome sequencing, and seeds were preserved at the School of Forestry, Northeast Forestry University, under the designation Int-OL-202204. In August 2022, roots, stems, leaves, flowers, and seeds of *I. noli-tangere* were collected for transcriptomic sequencing and metabolomic analysis. In addition, leaves were harvested from 8-week-old transgenic *N. tabacum* plants for transcriptomic and metabolomic analyses.

### Genome sequencing

Genomic DNA was extracted from young leaves of *I. noli-tangere* using the DNA Secure Plant Kit (Tiangen, China). After constructing an Illumina genomic library, paired-end sequencing was performed with the Illumina PE150 platform (2 × 150 bp). Additionally, CCS reads were obtained using the PacBio Sequel II platform. Finally, a Hi-C library was constructed following Belton's method and sequenced using the Illumina PE150 platform [47]. All sequencing services were provided by Novogene Co., Ltd., Beijing, China.

### Genome size assessment

The genome size was estimated by analyzing the frequency distribution of 17-kmers using Jellyfish software on high-quality clean reads. The frequency distribution is presented in Fig. S1. The genome size was calculated as follows: Genome size = K-mer number/K-mer Peak [48].

### Genome assembly

High-quality PacBio HiFi reads (42.02 Gb, 68.39× coverage depth) were generated using the Sequel II platform. Raw reads from PacBio, Hi-C (62.70 Gb, 102.04×), and Illumina platforms (37.96 Gb, 61.78×) underwent rigorous quality control via fastp (v0.23.2), which removed adapter-contaminated reads, reads with >10% ambiguous bases (N), and read pairs where >20% of bases in either read had Phred scores <5.

*De novo* assembly was performed using hifiasm (v0.16.1), leveraging its haplotype-resolving capability to produce a gap-free primary assembly. Contigs were subsequently polished with ≥10× coverage Illumina short reads using Pilon (v1.22), resulting in a final assembly with a base-level accuracy of QV ≈ 45.08, as estimated by k-mer analysis [49]. Hi-C data were preprocessed with HiCUP (v0.8.3) to filter invalid ligation products and retain high-quality interactions. Chromosome-scale scaffolding was performed with ALLHiC (v0.9.8), followed by manual curation in Juicebox (v2.2.6) to resolve misorientations and enhance topological consistency based on chromatin interaction patterns [50].

### Genome annotation

The protein-coding genes of the genome were annotated through a comprehensive approach that combined *ab initio*, transcriptome-assisted prediction, and homology-based prediction. For *ab initio* prediction, various software tools were utilized, including Augustus (v3.2.3), Geneid (v1.4), Genescan (v1.0), GlimmerHMM (v3.04), and SNAP (v2013-11-29) [51–53]. In addition, for homology-based predictions, protein sequences from *A. thaliana*, *C. oleifera*, and *I. glandulifera* were employed as query sequences, conducting a TblastN (v2.2.26) search against the reference genome with an *E*-value threshold of 1e−5 [54]. To enhance the annotation process, transcriptome-assisted prediction was performed by assembling RNA-seq data to reference transcripts using HISAT (v2.0.4) and StringTie (v1.3.3), while non-reference transcripts were assembled with Trinity (v2.11). Gene prediction was further refined using PASA (v2.0.2) [55]. The final non-redundant gene set was generated with EvidenceModeler (EVM) (v1.1.1), and the annotation results were evaluated using BUSCO [56]. For the identification of non-coding RNAs, Rfam (v14.1) and Infernal (v1.1.2) were utilized [57]. In order to determine the functional roles of the genes, functional annotation was performed using InterProScan (v5.31), aligning the findings with established databases such as PFAM and GO [58].

### Repeat sequence annotation

Repeat sequences in the genome were annotated using the Extensive de-novo TE Annotator (EDTA, v2.0.1) combined with the SINE Base database, and unannotated LTRs were re-annotated using DeepTE (v2020-12-25) [59, 60].

### Phylogenetic analysis and divergence time estimation

A total of 13 species were included in the phylogenetic analysis: *O. sativa*, *I. noli-tangere*, *I. glandulifera*, *C. lanceoleosa*, *A. chinensis*, *D. lotus*, *V. paradoxa*, *R. communis*, *H. annuus*, *C. canephora*, *S. indicum*, *S. hispanica*, and *A. thaliana*. While the genome of *I. noli-tangere* was sequenced and assembled in this study, the genomes of the other 12 species were sourced from public databases, with specific sources detailed in Table S10. Orthologous genes were identified and clustered using OrthoFinder (v2.3), and gene family expansion and contraction analyses were performed with the CAFE software [61, 62]. The phylogenetic tree was constructed using ASTRAL (v5.7.3), and divergence times were calculated with the MCMCTREE program in PAML (v4.5) [63, 64].

Calibration times were obtained from the TimeTree database (http://timetree.org/) [65], with key nodes cross-validated using fossil evidence and published literature [66–69]. Key calibrated nodes included the divergence between monocots and eudicots (142.1–163.5 Mya), core eudicot divergence (111.4–123.9 Mya), the split between Asterales and Ericales (101.5–115.6 Mya), the origin of Ericales (93.9–114.1 Mya), and the divergence between *I. noli-tangere* and *I. glandulifera* (8.47–27.21 Mya).

### Whole-genome duplication analysis and identification

Genomic collinearity was assessed through all-vs-all BLASTP (v2.2.26), with an *E*-value threshold set at 1e−5. Syntenic blocks were subsequently identified from these alignments using WGDI (v0.6.5), which was also employed to calculate *K*s [70]. To distinguish orthologous from paralogous syntenic blocks with higher precision, the SOI toolkit (https://github.com/zhangrengang/SOI) was employed with the Orthology Index set to 0.6 (default parameters retained). This threshold has been validated as broadly effective for orthologous syntenic block identification [71]. Resulting orthology-defined blocks were visualized in *K*s-colored syntenic dot plots. The phylogenetic placement of WGD events further examined using the MAPS algorithm, following the approach described by Li [72]. A reference species tree was constructed including *I. noli-tangere*, *C. lanceoleosa*, *A. chinensis*, *D. lotus*, and *V. vinifera*, with *O. sativa* as the outgroup. Gene families were identified using OrthoFinder based on BLASTP (*E*-value < 1e−5), and only those containing at least one gene copy from each taxon were retained. For each retained gene family, protein sequences of each gene family were aligned and phylogenetically analyzed using PASTA. The best-scoring PASTA tree for each multi-species nuclear gene family was used to estimate the number of shared gene duplications on each branch of the species tree. To evaluate whether these duplications exceeded background levels, null simulations were conducted by modeling gene birth and death across the species tree, with parameter estimates obtained from WGDgc. Fisher's exact tests were then applied to detect nodes with significantly elevated duplication frequencies relative to the null expectation. In addition, positive simulations assuming a WGD at internal nodes were performed to assess whether the observed duplication patterns were consistent with WGD scenarios.

### Genes involved in oil biosynthesis and analysis

To identify genes involved in oil biosynthesis, we first referred to the ARALIP database (http://aralip.plantbiology.msu.edu/), which provides curated information on *A. thaliana* genes involved in acyl-lipid metabolism. Genes from the ‘fatty acid synthesis and export’ and ‘triacylglycerol synthesis’ categories were extracted and mapped to the KEGG database. These genes were primarily assigned to the following KEGG pathways: ko00061 (fatty acid biosynthesis), ko00062 (fatty acid elongation), ko01040 (biosynthesis of unsaturated fatty acids), and M00089 (triacylglycerol biosynthesis, part of ko00561). In addition, genes encoding fatty acid desaturases were included due to their essential roles in determining the composition of unsaturated fatty acids. Based on this reference set, the protein sequences of *I. noli-tangere* were annotated by BLASTP alignment against the KEGG database (*E*-value < 1e−5), and genes assigned to the above pathways were identified as oil biosynthesis-related candidates [30].

These genes were further categorized into functional stages according to their corresponding KEGG modules. Genes involved in M00082 (fatty acid biosynthesis, initiation) were assigned to the initiation stage. Genes in M00415 (fatty acid elongation in endoplasmic reticulum), M00083 (fatty acid biosynthesis, elongation), and M00085 (fatty acid elongation in mitochondria) were classified under the elongation stage. Genes encoding fatty acid desaturases and thioesterases such as fatty acyl-ACP thioesterase and acyl-CoA thioesterase, were grouped into the modification stage. Genes involved in M00089 (triacylglycerol biosynthesis) were assigned to the assembly stage, while those in M00086 (beta-oxidation, acyl-CoA synthesis) and M00087 (beta-oxidation) were assigned to the degradation stage.

Gene duplication types in the *I. noli-tangere* genome were identified using DupGen_finder-unique [73]. According to the tool's documentation, the choice of outgroup primarily affects the identification of TRD genes. Selecting a closely related outgroup facilitates more accurate detection of TRD genes that emerged after divergence from the outgroup species. In this study, *I. glandulifera*, a closely related species, was chosen as the outgroup to enable more precise identification of TRD genes that arose during the independent evolution of *I. noli-tangere*. Additionally, the *K*a and *K*s rates, along with the *K*a/*K*s ratio, were calculated for each duplicated gene pair using the YN model implemented in KaKs_Calculator v2.0 [74].

### Transcriptome sequencing

Plant materials, including multiple organ samples from *I. noli-tangere* and transgenic *N. tabacum* leaves, were immediately flash-frozen in liquid nitrogen and stored at −80°C until RNA extraction. For each sample, 0.2 to 0.5 g of frozen tissue was finely ground in a liquid nitrogen-precooled mortar, lysed with TRIzol™ reagent, and subjected to chloroform-based phase separation. Total RNA was precipitated with isopropanol, washed with ethanol, and purified. RNA integrity was initially assessed by 1.2% agarose gel electrophoresis, which showed distinct 28S and 18S rRNA bands without genomic DNA contamination. Further quality assessment using an Agilent 5400 Bioanalyzer yielded RNA Integrity Number (RIN) values ranging from 7.7 to 9.8, confirming the RNA was of high quality and suitable for high-throughput sequencing. RNA sequencing was conducted on the Illumina NovaSeq 6000 platform with non-strand-specific paired-end libraries.

### Gene family analysis

Using *A. thaliana* bZIP and FAD family genes as reference sequences, the protein sequences of *I. noli-tangere* were compared using BLAST, with an *E*-value threshold of 1e−5. Additionally, based on Hidden Markov Model (HMM) domains from the Pfam database for bZIP transcription factors (PF00170) and fatty acid desaturases (PF03405 and PF00487), searches were conducted using HMMER software (v3.3.2) on *I. noli-tangere*. The results from BLAST and HMMER were combined, duplicates were removed, and a phylogenetic tree was constructed using IQ-TREE (v2.1.2) [75].

### Fatty acid component analysis

Fatty acid composition was analyzed following the procedures outlined in the Chinese national standard GB 5009.168-2016, with modifications based on the method described by Guan [76]. Lipids were extracted via diethyl ether, and undecanoic acid (C11:0, 1 mg) was added as an internal standard. The extracted lipids were then methylated by adding 2 ml of 14% boron trifluoride-methanol solution to a precisely weighed oil sample. The mixture was incubated at 60°C for 30 min in a water bath, cooled to room temperature, and subsequently extracted with 2 ml of n-hexane and 1 ml of distilled water. After phase separation, the upper organic layer was collected, evaporated to dryness under nitrogen, and redissolved in 1 ml of n-hexane for GC–MS analysis.

GC–MS analysis was performed using an Agilent 7890-5975 system equipped with an HP-5MS capillary column (60 m × 0.25 mm, 0.25 μm). The injection port was maintained at 280°C with a split ratio of 20:1, and helium was used as the carrier gas at a constant linear velocity of 1.5 ml/min. A 1.0 μl sample was injected. The oven temperature program was as follows: initial temperature of 120°C held for 1 min, increased at 6°C/min to 170°C, then at 2.5°C/min to 215°C and held for 12 min, followed by an increase at 4°C/min to 230°C and held for 10 min, and finally increased at 10°C/min to 280°C and held for 15 min.

Mass spectrometry was performed in electron impact mode at 70 eV, with the ion source temperature at 200°C, quadrupole temperature at 150°C, and interface temperature at 260°C. The mass scan range was m/z 40 to 550, with a solvent delay of 4.4 min. Fatty acid methyl esters (FAMEs) were identified by comparing their retention times and mass spectra with those of a 37-component FAME standard mixture, which served as the reference for compound identification. Quantitative analysis was performed using the internal standard method, with the relative peak areas of individual FAMEs normalized against that of C11:0.

### Plant transformation and quantitative RT-qPCR analysis

The CDS sequences of *IntFAD3*, *IntbZIP38*, and *Int13-HPL* (with stop codons removed) were cloned into the pBI121 vector. Using Agrobacterium-mediated transformation, these constructs were introduced into *N. tabacum*. The expression levels of the target genes in transgenic plants were analyzed with *NtACTIN* as the internal reference gene, screening for stable T2 generation transgenic tobacco lines. Primer information is provided in Table S11.

### Y1H assay

The CDS sequence of *IntbZIP38* was cloned into the pGADT7, and the sequences of *proIntFAD3-P1*, *proIntFAD3-P2*, and *proIntFAD3-P3* were fused into the pAbAi vector. Following the method of Pyvovarenko, a bait yeast strain was constructed, and the concentration of AbA was determined before transforming pGADT7-*IntbZIP38* into the bait strain to assess interactions [77]. The primers used are listed in Table S11.

### Dual-luciferase reporter assay

The CDS sequence of *IntbZIP38* was cloned into the pGreenII 62-SK to construct an effector expression vector, while the *proIntFAD3*, *proIntFAD3-fragment1* and *proIntFAD3-fragment2* sequences were inserted into the pGreenII 0800-LUC plasmid to construct a reporter gene vector. These plasmids were then transformed into GV3101 strains containing pSoup-p19 to prepare suspensions. According to the experimental design, the suspension was mixed and injected into the leaves of *N. benthamiana* for transient expression. Following Zhang's method, the transiently transformed *N. benthamiana* plants were cultivated and treated [78]. Chemiluminescent imaging was performed using a plant imaging system (Tanon 5200, Tanon, China), and the LUC/REN ratio was measured using a multifunctional microplate reader (SpectraMax iD3, Molecular Devices, USA). The primers used are listed in Table S11.

### GUS activity assay

The promoter fragments were separately cloned into the pCAMBIA1304 plasmid to construct GUS reporter gene vectors. The CDS sequence of *IntbZIP38* was cloned into the pCAMBIA1300 plasmid to construct an effector expression vector. Following Yuan's method, *N. benthamiana* was subjected to transient transformation, followed by GUS staining and quantification experiments [79]. Primer information is provided in Table S11.

### SPME-GC–MS

Modified from the method of Wang [80], 1 g of a sample was weighed and placed in a 20 ml headspace vial, to which 20 μl of isoamyl phenylacetate (0.05 mg/ml) was added as an internal standard. The vial was sealed and incubated in a water bath at 60°C with magnetic stirring at 500 rpm for 20 min. A Supelco 57 348-U SPME fiber, preconditioned in the GC inlet at 250°C for 20 min, was then inserted into the vial for solid-phase microextraction for 30 min. Volatile compounds were subsequently desorbed in the GC injection port and analyzed using an Agilent 6890N-5973 GC–MS system.

GC–MS analysis was performed with a HP-INNOWax capillary column (60 m × 250 μm × 0.25 μm). The injector temperature and the GC–MS interface temperature were both set at 250°C. The carrier gas (helium) was maintained at a constant flow rate of 1.5 ml/min, with a split ratio of 4:1. The oven temperature program was as follows: initially held at 40°C for 5 min, ramped at 5°C/min to 250°C, and held at 250°C for 10 min. The mass spectrometer operated in electron ionization mode at 70 eV, with an ion source temperature of 230°C, quadrupole temperature of 150°C, and a scan range of m/z 35 to 550. Volatile compounds were quantified using the internal standard method. For tobacco samples, (E)-2-hexenal was quantified using a calibration curve established with (E)-2-hexenal standard and the internal standard.

### Essential oil extraction and analysis

Following Liu's method, essential oils were extracted from the stems and leaves of *I. noli-tangere* using microwave-assisted extraction [81]. The extracted 50 mg of essential oil was dissolved in 5 ml of n-hexane, dehydrated, and filtered. Component analysis was conducted using the Agilent 7890A-7000B GC–MS system following Peng's method [82].

### Antifungal experiment

The strain of *B. cinerea* was obtained from the Agricultural Culture Collection of China (ACCC), with the strain collection number ACCC-37356. The spore suspension was prepared following Li's method, and the MIC was determined using the broth microdilution method and the resazurin reduction test [83]. To avoid cross-interference between samples of different concentrations caused by essential oil volatilization, each sample was individually processed in a 500 μl centrifuge tube. After staining, the supernatant was aspirated and placed onto a cell culture plate for imaging and statistical analysis. Mycelial growth measurements were conducted based on Yan's approach, where essential oils and standards were dissolved in 1 ml of 0.5% Tween-80 solution and added to a PDA medium to achieve the desired concentrations [84]. A negative control using 0.5% Tween-80 was set, and 5 mm mycelial plugs taken from the edges of 7-day-old *B. cinerea* mycelia were inverted onto the center of PDA plates. These plates were incubated at 25 ± 2°C for 7 days, and images were taken for statistical analysis.

## Supplementary Material

Web_Material_uhaf216

## Data Availability

The genome assembly has been deposited in the China National GeneBank DataBase (CNGBdb, https://db.cngb.org/), under accession number CNP0005329. Transcriptomic sequencing data are available at the NCBI database (https://www.ncbi.nlm.nih.gov/) under accession number PRJNA1131936 and PRJNA1257970. The GenBank accession numbers for the nucleotide sequences utilized in this study are as follows: *IntFAD3* (PQ570564), *IntFAD7* (PQ570566), *IntbZIP38* (PQ570565), *Int13-HPL* (PQ570563).
