# Supplementary material for: High-quality genome assembly of Impatiens noli-tangere reveals key insights into α-linolenic acid biosynthesis and metabolic volatiles
Source: Hortic Res. 2025 Aug 22;12(11):uhaf216. doi: 10.1093/hr/uhaf216 (PMC12598466; doi:10.1093/hr/uhaf216)
Supplement: Web_Material_uhaf216 [file web_material_uhaf216.zip › Figure S10. Comparison of ALA percentage in the leaf fatty acid composition between transgenic N. tabacum and empty vector control.pdf]

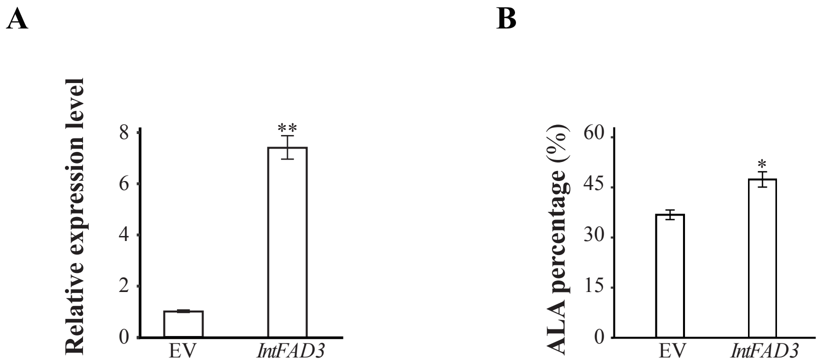

Figure S10. Comparison of ALA percentage in the leaf fatty acid composition between transgenic *N. tabacum* and empty vector control.
A Relative expression levels of *IntFAD3* in empty vector (EV) and transgenic *N. tabacum*.
B ALA percentage in the leaf fatty acid composition of transgenic *N. tabacum*.
