# Supplementary material for: High-quality genome assembly of Impatiens noli-tangere reveals key insights into α-linolenic acid biosynthesis and metabolic volatiles
Source: Hortic Res. 2025 Aug 22;12(11):uhaf216. doi: 10.1093/hr/uhaf216 (PMC12598466; doi:10.1093/hr/uhaf216)
Supplement: Web_Material_uhaf216 [file web_material_uhaf216.zip › Figure S11. Pearson correlation analysis between IntbZIP family genes and IntFAD3 expression levels.pdf]

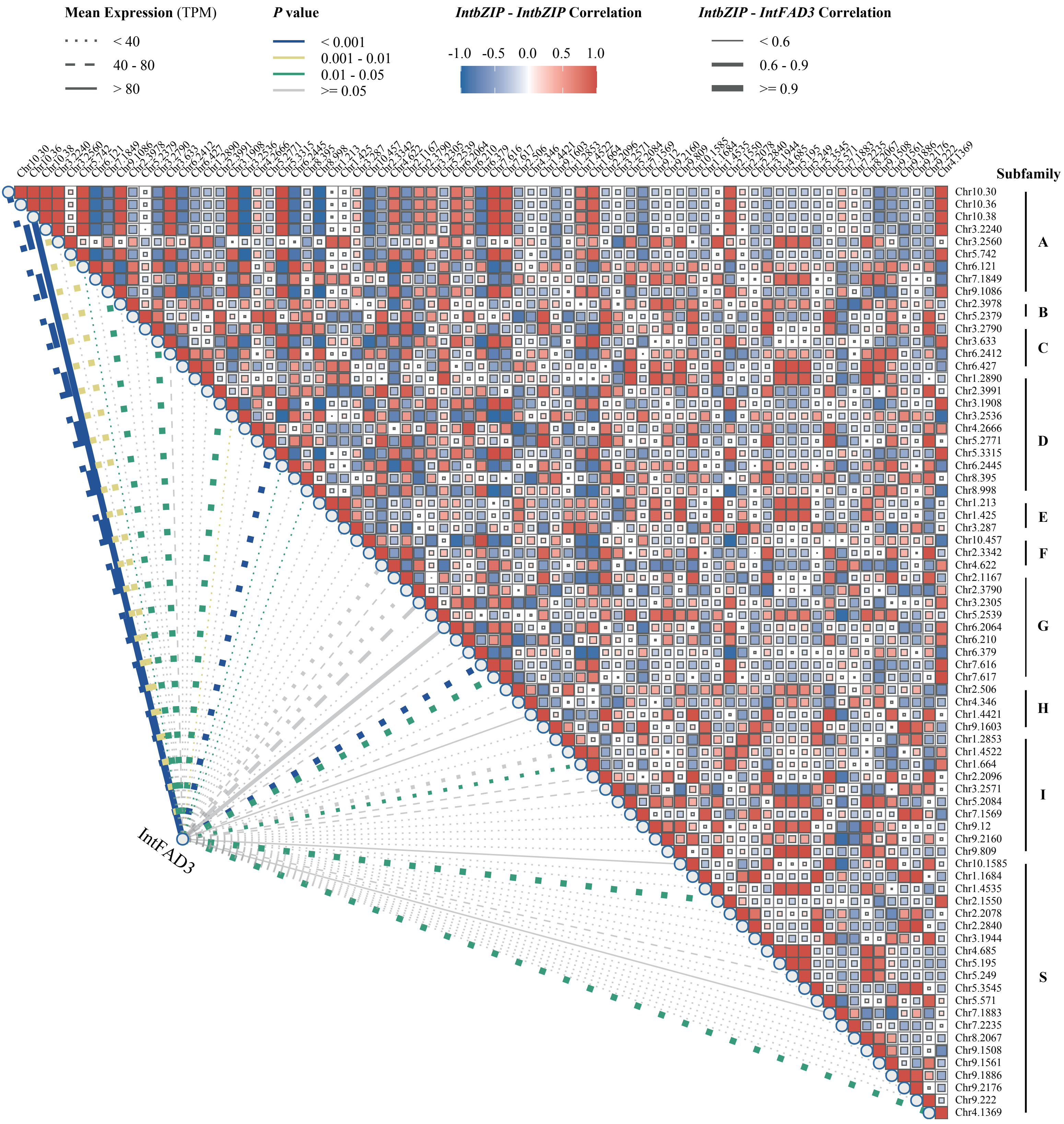

Figure S11. Pearson correlation analysis between *IntbZIP* family genes and *IntFAD3* expression levels. Gene family analysis was performed based on the neighbor-joining algorithm.
