# Supplementary material for: High-quality genome assembly of Impatiens noli-tangere reveals key insights into α-linolenic acid biosynthesis and metabolic volatiles
Source: Hortic Res. 2025 Aug 22;12(11):uhaf216. doi: 10.1093/hr/uhaf216 (PMC12598466; doi:10.1093/hr/uhaf216)
Supplement: Web_Material_uhaf216 [file web_material_uhaf216.zip › Figure S14. Effects of essential oils and their main compounds from the stem and leaf of I. noli-tangere on B. cinerea mycelial growth.pdf]

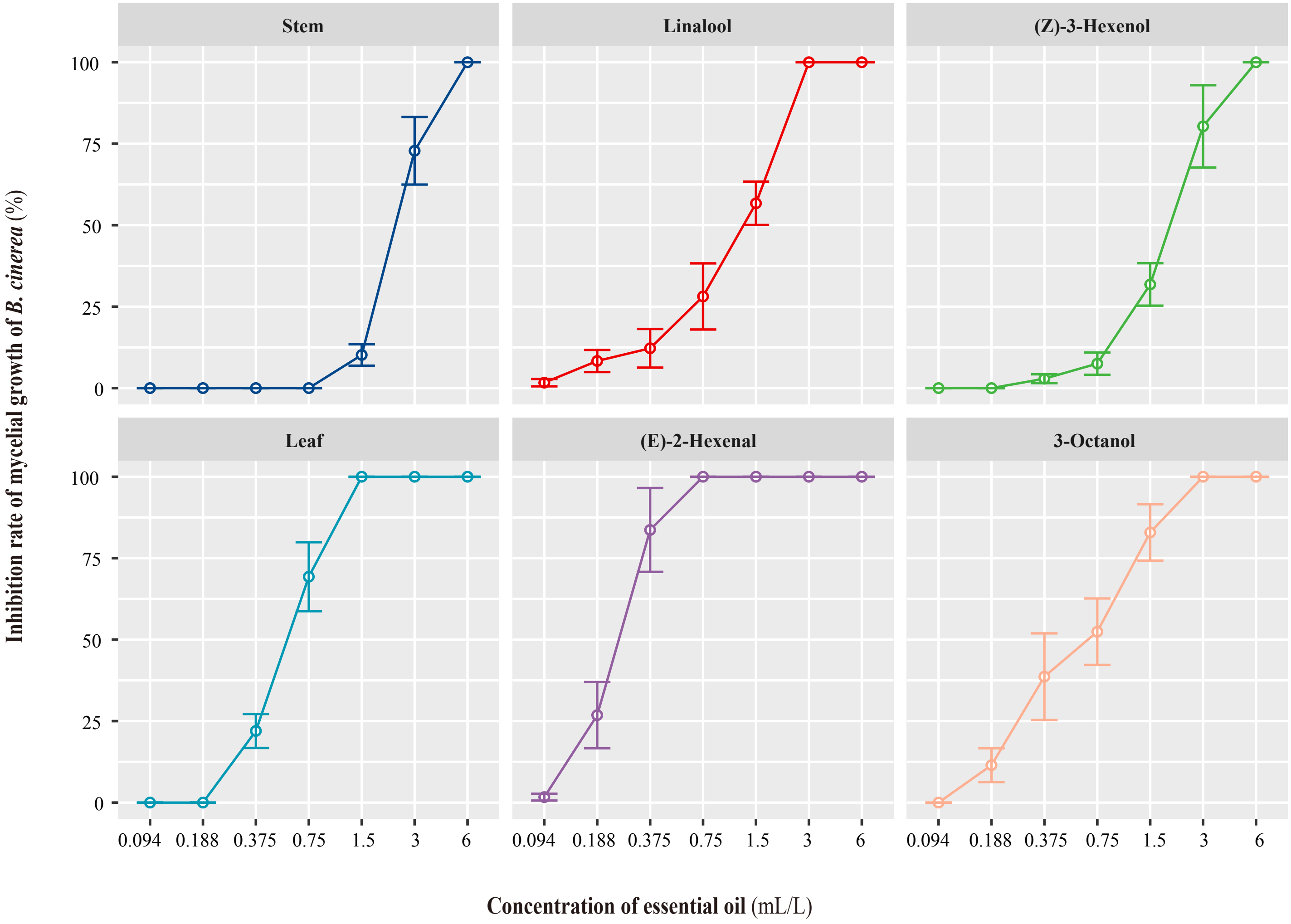

Figure S14. Effects of essential oils and their main compounds from the stem and leaf of *I. noli-tangere* on *B. cinerea* mycelial growth.
