# Supplementary material for: High-quality genome assembly of Impatiens noli-tangere reveals key insights into α-linolenic acid biosynthesis and metabolic volatiles
Source: Hortic Res. 2025 Aug 22;12(11):uhaf216. doi: 10.1093/hr/uhaf216 (PMC12598466; doi:10.1093/hr/uhaf216)
Supplement: Web_Material_uhaf216 [file web_material_uhaf216.zip › Figure S15. Fungicidal activity of (Z)-3-Hexenol.pdf]

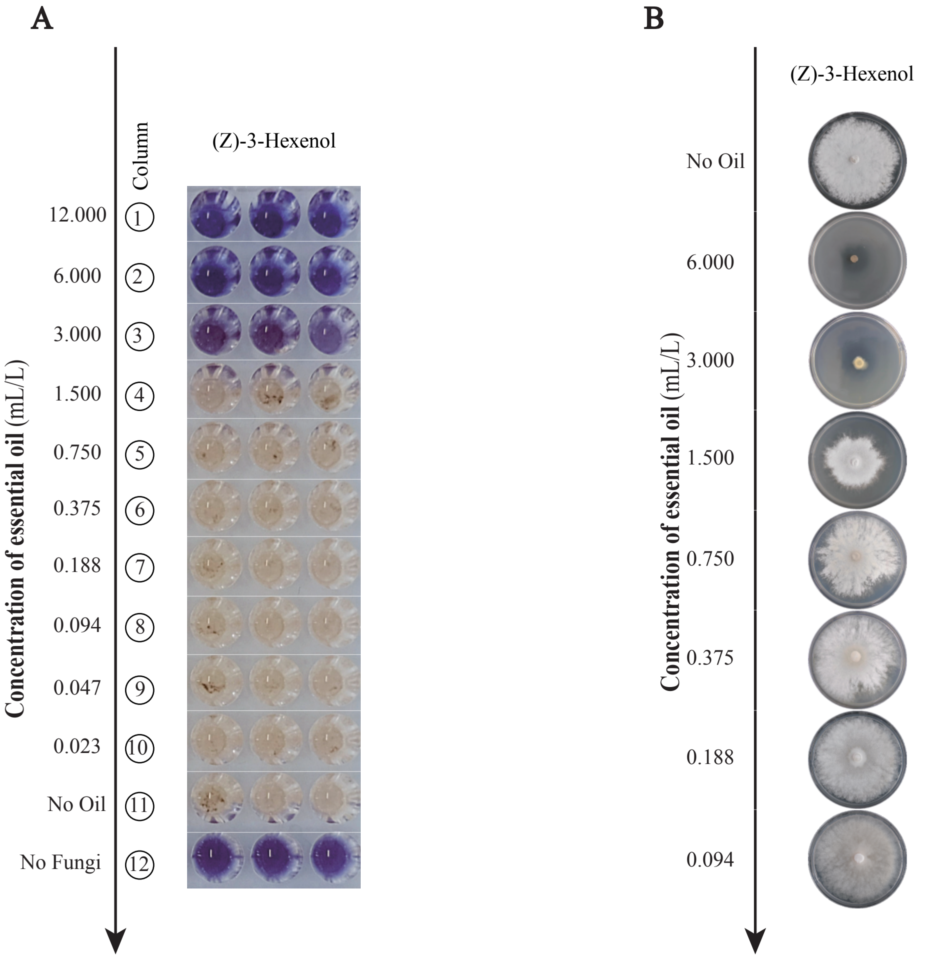

Figure S15. Fungicidal activity of (Z)-3-Hexenol.

A MIC of (Z)-3-Hexenol against *B. cinerea* determined by microdilution methods.

B Mycelium growth of *B. cinerea* treated with 0 (ck), 0.094, 0.188, 0.375, 0.75, 1.5, 3, and 6 mL/L of (Z)-3-Hexenol.
