# Supplementary material for: High-quality genome assembly of Impatiens noli-tangere reveals key insights into α-linolenic acid biosynthesis and metabolic volatiles
Source: Hortic Res. 2025 Aug 22;12(11):uhaf216. doi: 10.1093/hr/uhaf216 (PMC12598466; doi:10.1093/hr/uhaf216)
Supplement: Web_Material_uhaf216 [file web_material_uhaf216.zip › Figure S4. Functional enrichment analysis of genes shared between I. noli-tangere with oil plants (R. communis, H. annuus, S. indicum, S. hispanica).pdf]

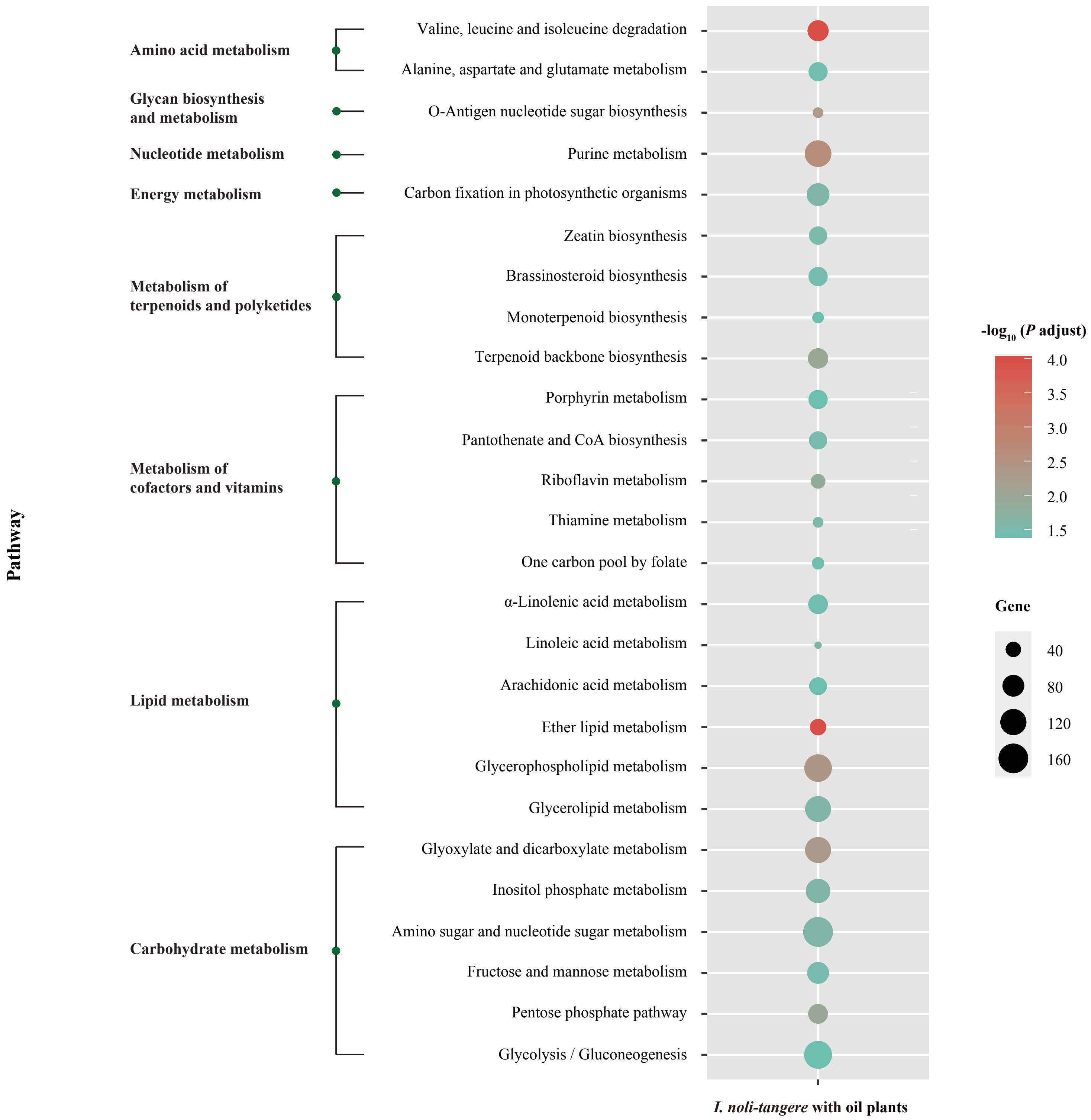

Figure S4. Functional enrichment analysis of genes shared between *I. noli-tangere* with oil plants (*R. communis*, *H. annuus*, *S. indicum*, *S. hispanica*). The enriched KEGG terms with corrected *P* adjust <0.05 are presented. The color of circles represents the statistical significance of enriched KEGG terms. The size of the circles represents the number of genes in a KEGG term. For all annotated genes their KEGG terms are provided as background information. '*P* adjust' is the Benjamini-Hochberg false discovery rate (FDR) adjusted *P* value.
