# Supplementary material for: High-quality genome assembly of Impatiens noli-tangere reveals key insights into α-linolenic acid biosynthesis and metabolic volatiles
Source: Hortic Res. 2025 Aug 22;12(11):uhaf216. doi: 10.1093/hr/uhaf216 (PMC12598466; doi:10.1093/hr/uhaf216)
Supplement: Web_Material_uhaf216 [file web_material_uhaf216.zip › Figure S5. Synteny relationships and Ks-based evidence for WGD events in I. noli-tangere, I. glandulifera, and C. lanceoleosa.pdf]

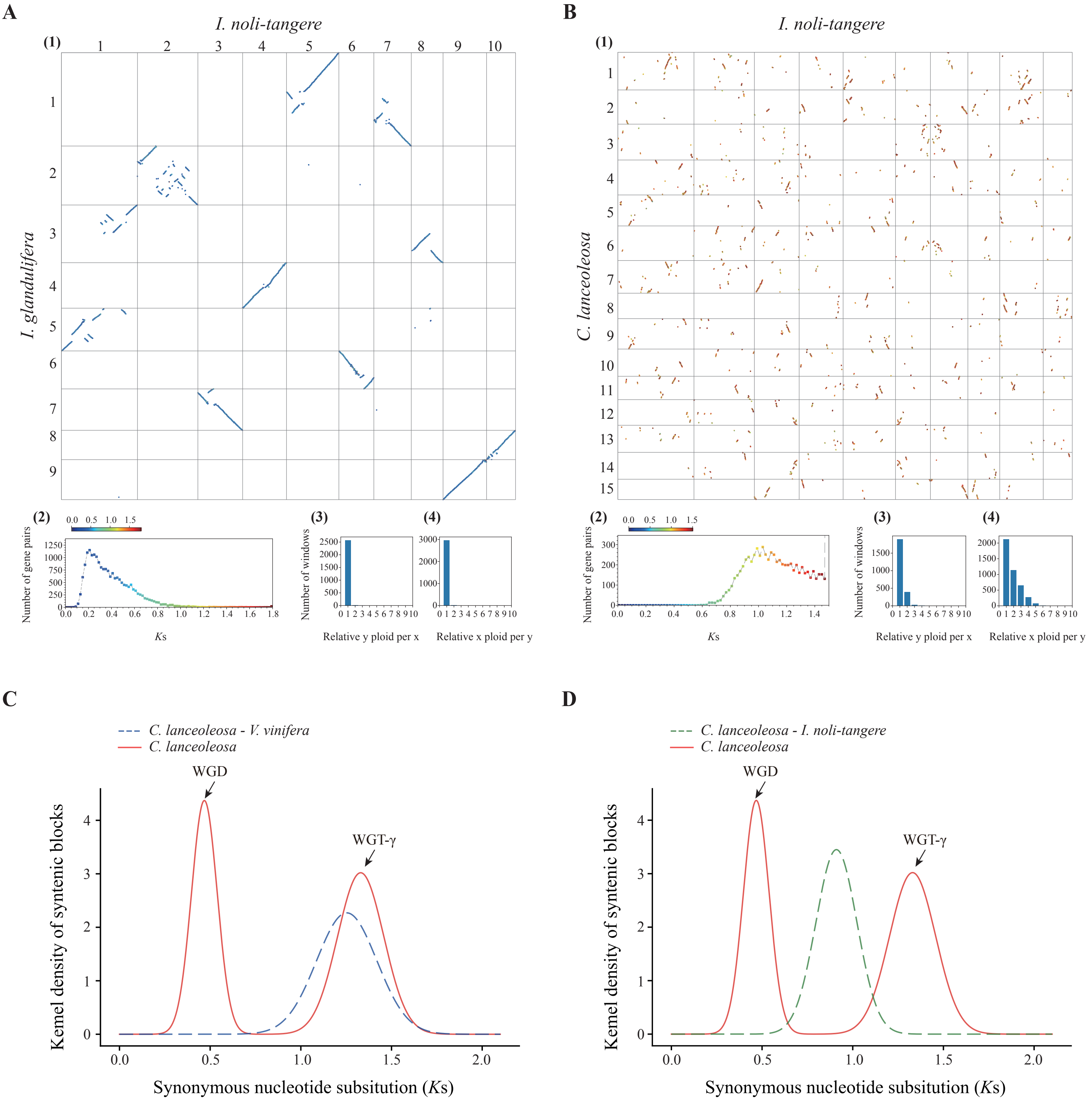

Figure S5. Synteny relationships and *K*s-based evidence for WGD events in *I. noli-tangere*, *I. glandulifera*, and *C. lanceoleosa*.

A, B *K*s-colored dot plots of orthologous synteny after applying an Orthology Index cutoff of 0.6. The plots illustrate a clear one-to-one orthology between *I. noli-tangere* and *I. glandulifera*, and a clear four-to-two orthology between *I. noli-tangere* and *C. lanceoleosa*.

(1) Dot plots color-coded by *K*s, with each dot representing an orthologous gene pair between the two genomes.

(2) Corresponding *K*s histograms using the same color scale as the dot plots.

(3-4) Orthologous synteny depth across 50-gene windows.

C, D *K*s distributions for paralogous genes within *C. lanceoleosa* and for orthologous genes between *C. lanceoleosa* and each of *V. vinifera* and *I. noli-tangere*.
