# Supplementary material for: High-quality genome assembly of Impatiens noli-tangere reveals key insights into α-linolenic acid biosynthesis and metabolic volatiles
Source: Hortic Res. 2025 Aug 22;12(11):uhaf216. doi: 10.1093/hr/uhaf216 (PMC12598466; doi:10.1093/hr/uhaf216)
Supplement: Web_Material_uhaf216 [file web_material_uhaf216.zip › Figure S6. KEGG enrichment analysis of overlapping genes among significantly expanded genes and the five types of duplicated genes (WGD, TD, PD, TRD, and DSD).pdf]

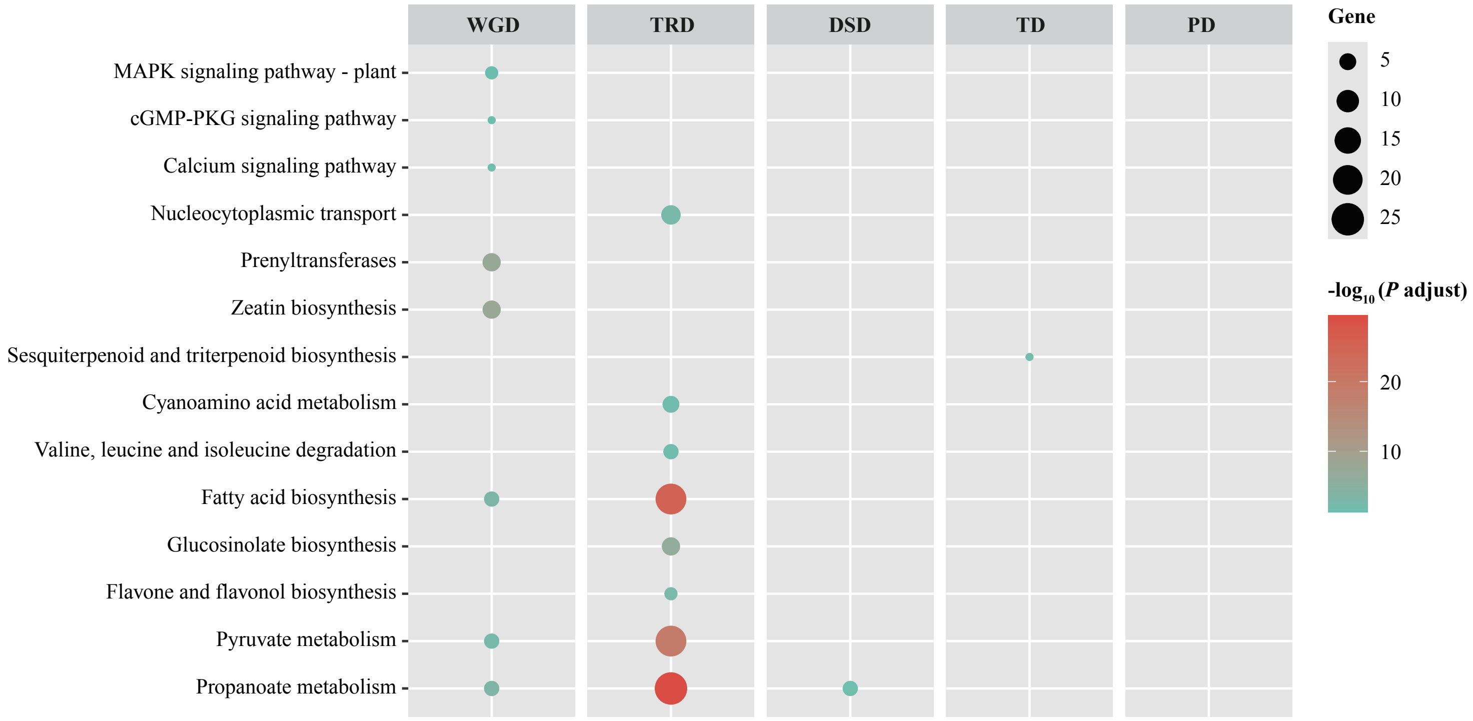

Figure S6. KEGG enrichment analysis of overlapping genes among significantly expanded genes and the five types of duplicated genes (WGD, TD, PD, TRD, and DSD). The figure shows all KEGG entries with $P < 0.05$. The size of the circles indicates the number of genes in each KEGG entry, while the color represents significance.
