# Supplementary material for: High-quality genome assembly of Impatiens noli-tangere reveals key insights into α-linolenic acid biosynthesis and metabolic volatiles
Source: Hortic Res. 2025 Aug 22;12(11):uhaf216. doi: 10.1093/hr/uhaf216 (PMC12598466; doi:10.1093/hr/uhaf216)
Supplement: Web_Material_uhaf216 [file web_material_uhaf216.zip › Figure S7. KEGG enrichment analysis of PD-derived genes.pdf]

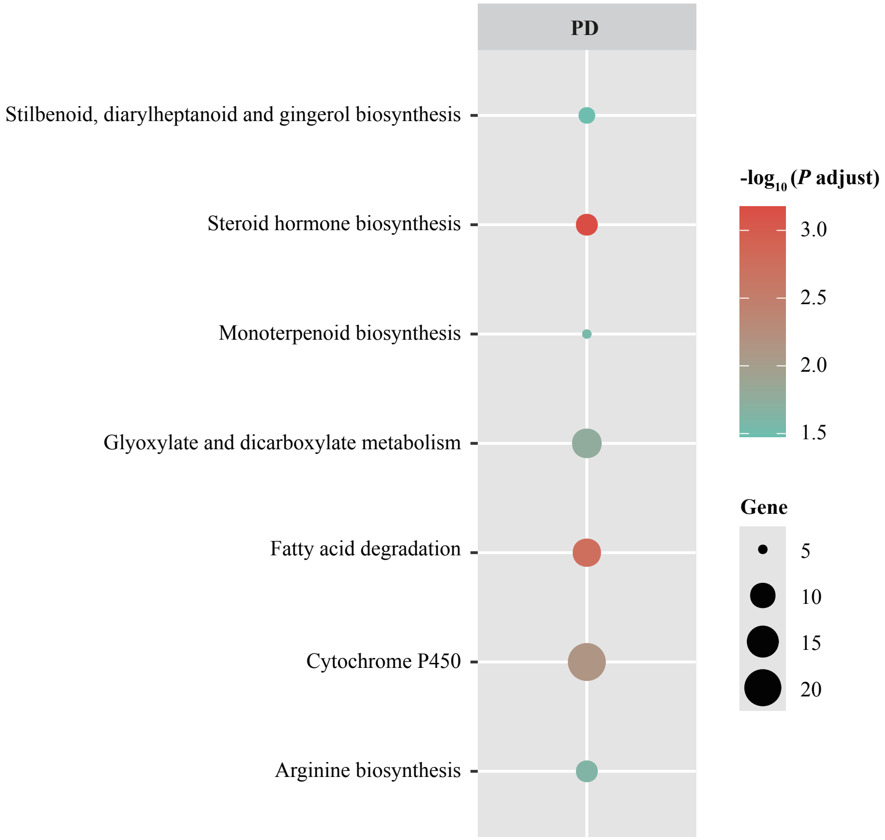

Figure S7. KEGG enrichment analysis of PD-derived genes. The figure presents all KEGG entries with $P < 0.05$. The size of the circles indicates the number of genes in each KEGG entry, while the color represents significance.
