# Supplementary material for: High-quality genome assembly of Impatiens noli-tangere reveals key insights into α-linolenic acid biosynthesis and metabolic volatiles
Source: Hortic Res. 2025 Aug 22;12(11):uhaf216. doi: 10.1093/hr/uhaf216 (PMC12598466; doi:10.1093/hr/uhaf216)
Supplement: Web_Material_uhaf216 [file web_material_uhaf216.zip › Figure S8. Phylogenetic tree of FAD genes in I. noli-tangere, and A. thaliana based on the neighbour-joining algorithm.pdf]

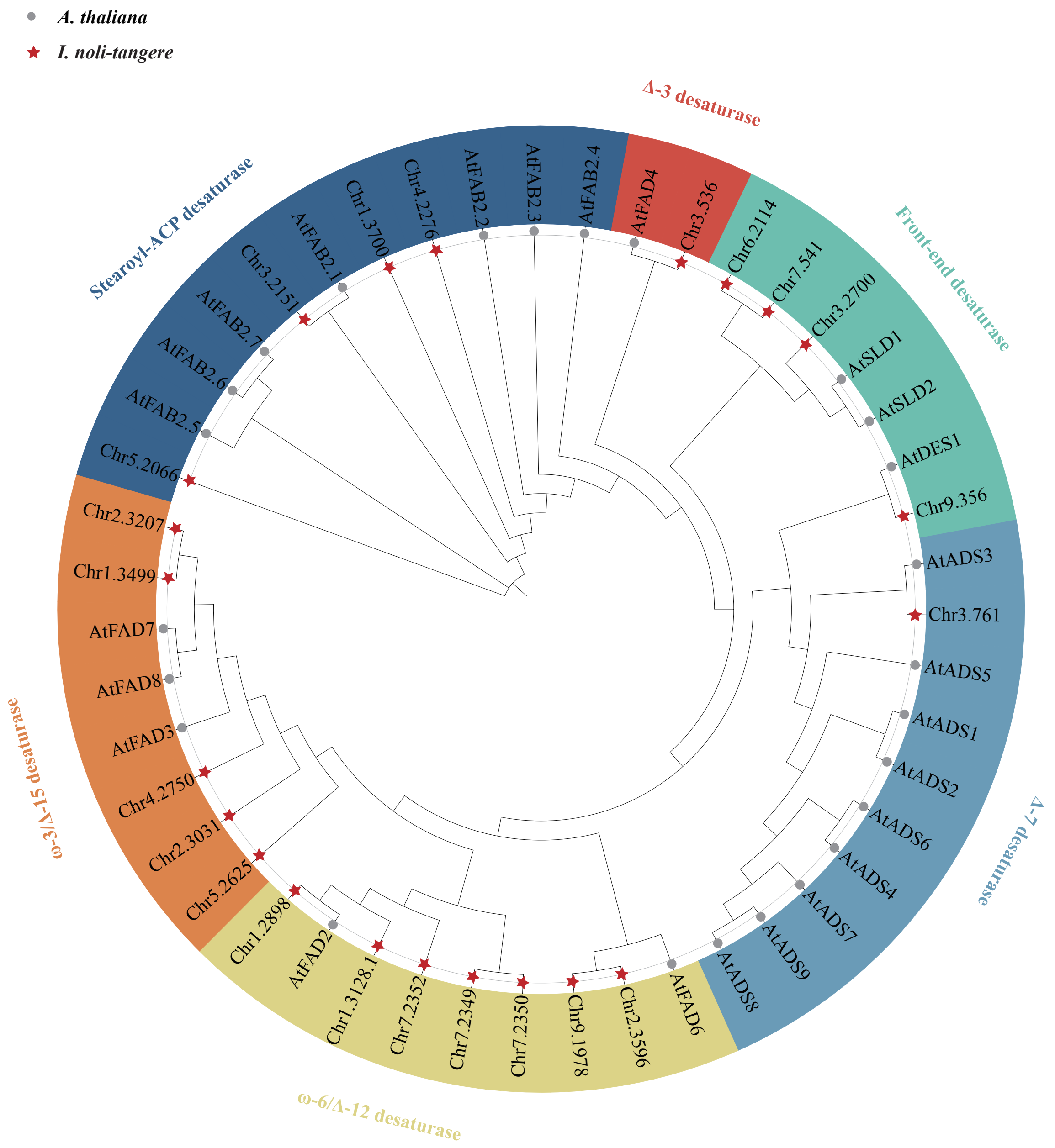

Figure S8. Phylogenetic tree of *FAD* genes in *I. noli-tangere*, and *A. thaliana* based on the neighbour-joining algorithm.
