# Supplementary figures and images for: High-quality genome assembly of Impatiens noli-tangere reveals key insights into α-linolenic acid biosynthesis and metabolic volatiles
Source: Hortic Res. 2025 Aug 22;12(11):uhaf216. doi: 10.1093/hr/uhaf216 (PMC12598466; doi:10.1093/hr/uhaf216)

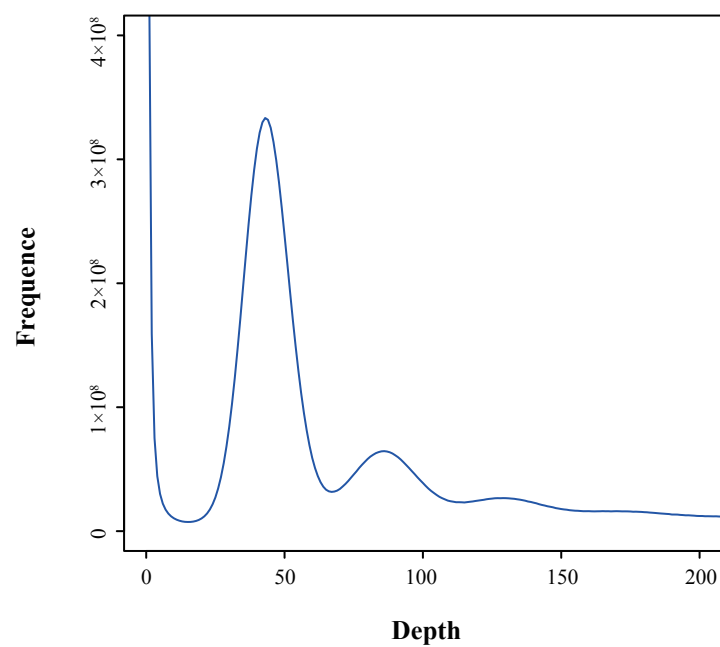

**Figure S1.** Distribution of 17-mer frequency in the *I. noli-tangere* genome.

Supplement: Web_Material_uhaf216 [file web_material_uhaf216.zip › Figure S1. Distribution of 17-mer frequency in the I. noli-tangere genome.pdf]

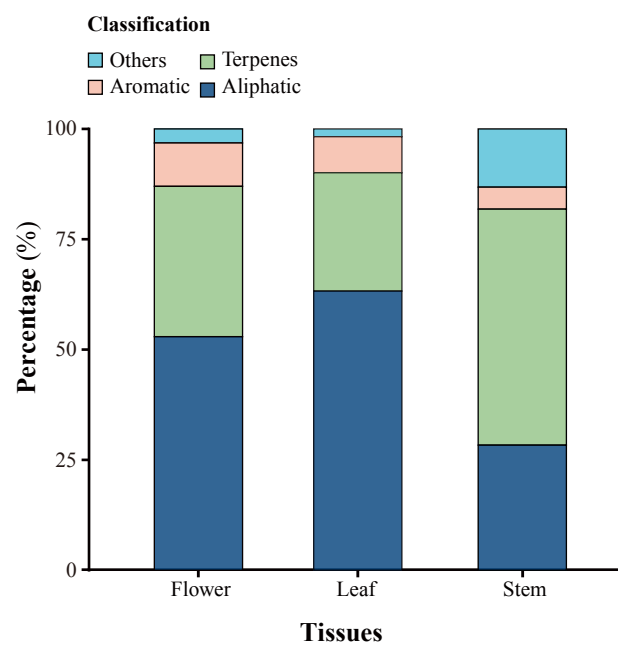

**Figure S12.** Volatile composition in the flower, leaf, and stem of *I. noli-tangere*.

Supplement: Web_Material_uhaf216 [file web_material_uhaf216.zip › Figure S12. Volatile composition in the flower, leaf, and stem of I. noli-tangere.pdf]

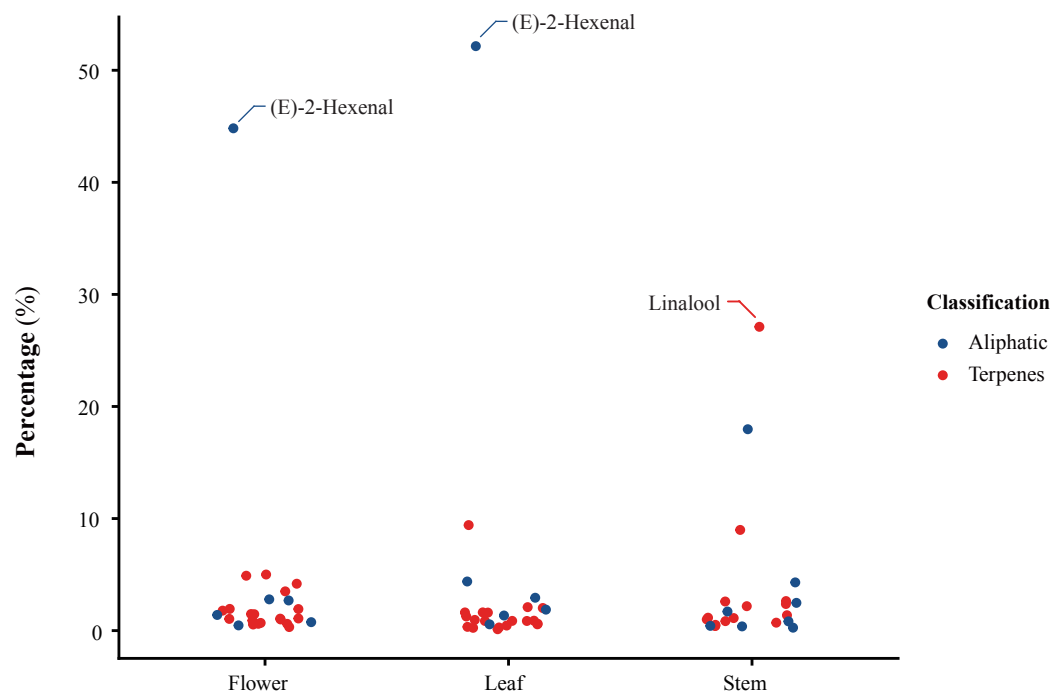

**Figure S13.** Terpene and aliphatic composition in the volatiles of the flower, leaf, and stem.

Supplement: Web_Material_uhaf216 [file web_material_uhaf216.zip › Figure S13. Terpene and aliphatic components in the volatiles of flower, leaf, and stem.pdf]

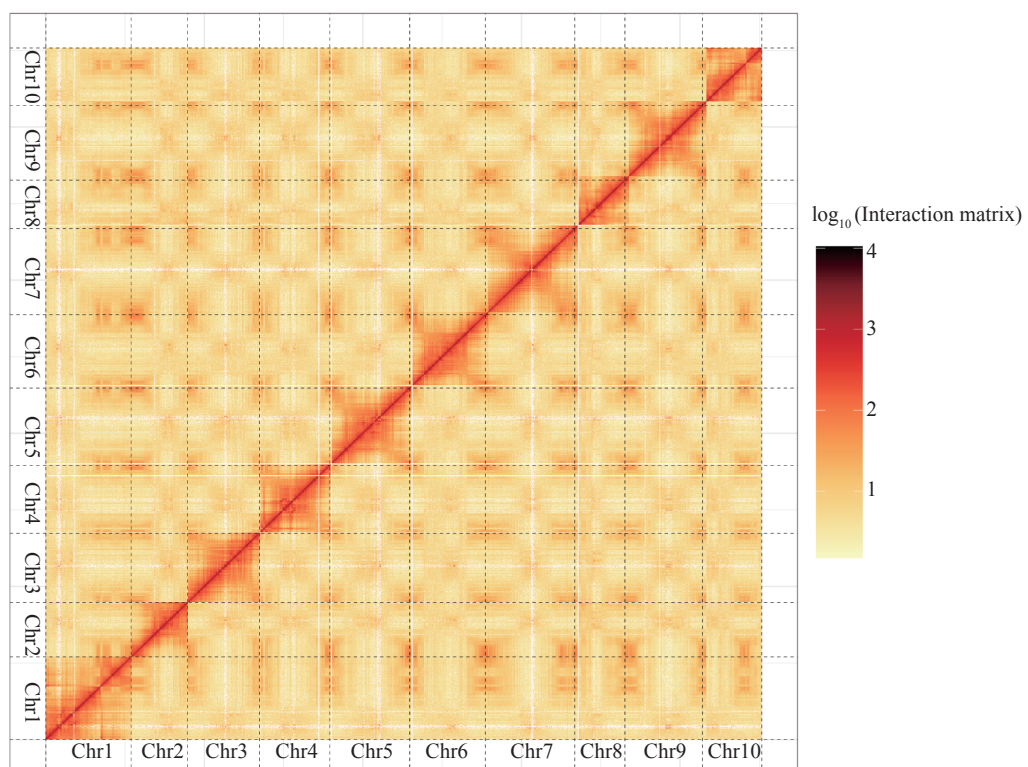

**Figure S2.** Hi-C interaction of the assembled *I. noli-tangere* genome.

Supplement: Web_Material_uhaf216 [file web_material_uhaf216.zip › Figure S2. Hi-C interaction of the I. noli-tangere assembled genome.pdf]
